# Drugs for the prevention and treatment of COVID-19 and its complications: An update on what we learned in the past 2 years

**DOI:** 10.3389/fphar.2022.987816

**Published:** 2022-10-10

**Authors:** Giuseppe Remuzzi, Stefano Schiaffino, Maria Gabriella Santoro, Garret A. FitzGerald, Gennaro Melino, Carlo Patrono

**Affiliations:** ^1^ Istituto di Ricerche Farmacologiche Mario Negri IRCCS, Bergamo, Italy; ^2^ Venetian Institute of Molecular Medicine (VIMM), Padova, Italy; ^3^ Department of Biology, University of Rome Tor Vergata, Rome, Italy; ^4^ Institute of Translational Pharmacology, CNR, Rome, Italy; ^5^ Institute for Translational Medicine and Therapeutics, Perelman School of Medicine, University of Philadelphia, Philadelphia, PA, United States; ^6^ Department of Experimental Medicine, University of Rome Tor Vergata, Rome, Italy; ^7^ Department of Pharmacology, Catholic University of the Sacred Heart, Rome, Italy

**Keywords:** drugs, biologics, COVID-19, treatment, prevention, cell therapy

## Abstract

The COVID-19 Committee of the Lincei Academy has reviewed the scientific evidence supporting the efficacy and safety of existing and new drugs/biologics for the preventing and treating of COVID-19 and its complications. This position paper reports what we have learned in the field in the past 2 years. The focus was on, but not limited to, drugs and neutralizing monoclonal antibodies, anti-SARS-CoV-2 agents, anti-inflammatory and immunomodulatory drugs, complement inhibitors and anticoagulant agents. We also discuss the risks/benefit of using cell therapies on COVID-19 patients. The report summarizes the available evidence, which supports recommendations from health authorities and panels of experts regarding some drugs and biologics, and highlights drugs that are not recommended, or drugs for which there is insufficient evidence to recommend for or against their use. We also address the issue of the safety of drugs used to treat underlying concomitant conditions in COVID-19 patients. The investigators did an enormous amount of work very quickly to understand better the nature and pathophysiology of COVID-19. This expedited the development and repurposing of safe and effective therapeutic interventions, saving an impressive number of lives in the community as well as in hospitals.

## 1 Introduction

Coronaviruses (CoV), a group of enveloped positive-strand RNA viruses, were discovered in the 1960s and originally thought to cause only mild disease in humans, with several strains found to be responsible for the common cold (Cui et al., 2019). This view changed in 2003 with the SARS (Severe Acute Respiratory Syndrome) pandemic and in 2012 with the MERS (Middle East Respiratory Syndrome) outbreak, two zoonotic infections that resulted in mortality rates above 10 and 35%, respectively (Fung and Liu, 2019).

The SARS-CoV-2 coronavirus was discovered by the end of 2019 and labelled as a public health emergency by the WHO in January 2020; the COVID-19 pandemic has now spread to about 600 million cases and caused about 6.5 million deaths (Johns Hopkins CSSE, 2021). Given the unprecedented impact of the pandemic in many countries, and the rise in the associated global death toll, over the past 2 years we have witnessed a race to find drugs/biologic treatments to save the lives of hospitalized, severely ill patients, as well as to develop vaccines. To this end, randomized clinical trials are underway to test experimental drug candidates and repurposed medicines. Therapeutic approaches to the early, mild phase of COVID-19 are also being debated and here, too, there is an emphasis on the need for randomized clinical trials. However, at times like these, regulatory authorities occasionally issue emergency use authorizations (EUAs) for drugs, as the United States Food and Drug Administration (FDA) initially did for chloroquine and hydroxychloroquine for COVID-19. The documentation supporting this FDA authorization, however, did not report or cite specific trials on which this decision was based, making it difficult to assess the scientific rationale. Nonetheless, physicians and healthcare providers interpreted the EUA for hydroxychloroquine as a directive to incorporate this drug into therapeutic protocols for treating COVID-19 patients. However, on June 15th the FDA announced that it was revoking the EUAs for these two drugs, explaining that they are “unlikely to be effective” and that current national treatment guidelines do not recommend using them outside of clinical trials (see Section 9 below).

Indeed, rigorous studies of COVID-19 drug candidates are fundamental to discriminating between anecdotes and reliable evidence for decisions on treatment. Otherwise, there is a high risk of sowing confusion among physicians caring for COVID-19 patients in these high-pressure situations.

Working Group 1a of the COVID-19 Committee of the Lincei Academy has previously prepared a brief review of the available scientific evidence regarding the efficacy and safety of existing and new drugs for the prevention and treatment of COVID-19 and its complications. This has now been updated to include what we learned in the past 2 years. Our focus is on drugs and neutralizing monoclonal antibodies that prevent the entry of SARS-CoV-2 into target cells; evidence on the benefits of the new drugs that inhibit SARS-CoV-2 replication; evidence of the risks/benefits of using non-steroidal anti-inflammatory drugs (NSAIDs); the place of corticosteroids in the treatment of critically ill patients with severe pulmonary complications of SARS-CoV-2 infection; evidence of the efficacy of immunomodulatory drugs, including anti-IL-6 receptor antagonists and Janus kinase (JAK) inhibitors; whether complement inhibitors, as well as anticoagulants and other antithrombotic agents have a place in the prevention and/or treatment of inflammatory and vascular complications of the disease; and on drugs that are not recommended because of a proven lack of efficacy, and drugs with insufficient evidence either way. Moreover, this updated review discusses the risks/benefits of using cell therapies, in particular mesenchymal stromal cells; presents evidence of the safety of concomitant medications prescribed for underlying conditions in COVID-19 patients; and reports how treatment was managed for children with Multisystem Inflammatory Syndrome (MIS-C), an illness temporally associated with preceding SARS-CoV-2 exposure.

This report does not intend to recommend any experimental drugs, but reviews the evidence supporting the efficacy and safety of these pharmacological treatments, highlights the official position of health authorities and panels of experts on each drug or class of drugs considered, and briefly mentions the ongoing trials registered with clinicaltrials.gov or the WHO registry.

## 2 Drugs and neutralizing monoclonal antibodies preventing SARS-CoV-2 entry into target cells

**FIGURE 1:**
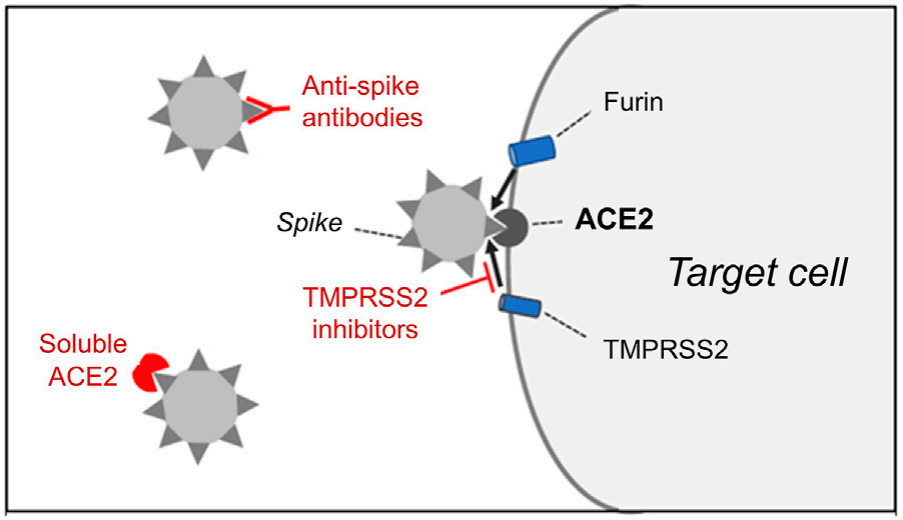
The entry of SARS-CoV-2 into target cells requires the binding of the spike protein present on the surface of the virus to cell membrane-bound angiotensin-converting enzyme 2 (ACE2), which acts as a receptor for the virus. Spike protein cleavage by the host cell proteases, transmembrane protease serine 2 (TMPRSS2) and furin, is also required for the fusion of the viral membrane with the plasma membrane of the cell. Ongoing clinical trials aim to determine whether cell entry of the virus can be prevented by 1) anti-spike antibodies, 2) soluble ACE2 extracellular domain or 3) inhibitors of TMPRSS2.

SARS-CoV-2 spike protein binds to its receptor, angiotensin-converting enzyme 2 (ACE2), and is proteolytically activated by the transmembrane protease serine 2 (TMPRSS2), thus enabling the fusion of the virus with the cell membrane (Hoffmann et al., 2020a; Zhou et al., 2020) (Figure 1). Bioinformatics analyses based on protein structures predict that transmembrane dipeptidyl peptidase-4 (DPP4), which is the receptor for MERS-CoV, could also interact with SARS-CoV-2 (Li et al., 2020). However, DPP4 was unable to mediate virus entry into cells lacking ACE2 (Zhou et al., 2020). Another tissue protease, the proprotein convertase furin, is involved in the cleavage of the spike protein, possibly promoting the subsequent cleavage by TMPRSS2 (Hoffmann et al., 2020b). However, furin inhibitors, unlike TMPRSS2 inhibitors, can interfere with important cell functions, so furin is not an attractive drug target. Current approaches that aim to block SARS-CoV-2 cell entry are based on 1) treatments that inhibit the SARS-CoV-2 spike-ACE2 interaction or 2) TMPRSS2 inhibitors.

Anti-spike antibodies and soluble ACE2 can block the interaction between the virus spike protein and ACE2. Passive immunization with convalescent plasma has been used in several countries to treat COVID-19, based on the rationale that neutralizing antibodies could both inhibit the virus from binding of the virus to the cell and promote immune cells clearance of the virus. Neutralizing antibodies are thus promising candidates for prophylactic and therapeutic treatment of COVID-19. Experience with other viral diseases indicates that donors with high serum titers of neutralizing antibody should be identified (a proportion of those who recover from COVID-19 have low titers) and the risk of antibody-dependent enhancement of infection (ADE) considered (Casadevall and Pirofski, 2020). Randomized clinical trials are needed to evaluate the efficacy and safety of anti-SARS-CoV-2 convalescent plasma, and at least two such trials are ongoing (*EudraCT Number: 2020–001310–38; ChiCTR Number: ChiCTR2000030010*). The recently updated NIH guidelines (https://www.covid19treatmentguidelines.nih.gov/) actually recommend against using convalescent plasma to treat COVID-19 in hospitalized patients without impaired humoral immunity. For non-hospitalized, immunocompetent COVID-19 patients, data from well-designed clinical trials are conflicting. Some demonstrate efficacy (Libster et al., 2021; Sullivan et al., 2022), while others have found no benefits (Korley et al., 2021; Alemany et al., 2022) regarding the incidence of disease progression and/or hospitalization. Notably, there is insufficient evidence for or against the use of high-titer convalescent plasma collected after the emergence of the Omicron variants. Monoclonal antibodies against SARS-CoV-2, some of which were derived from COVID-19 patients’ B-cells, were found to neutralize the virus in cultured cells (Wang C. et al., 2020; Andreano et al., 2021) and a study using a transgenic mouse model bearing human ACE2 confirmed that specific monoclonal antibodies can reduce virus titers in infected lungs (Wu et al., 2020). The latter report was complemented by a detailed structural analysis of the interaction between antibodies, the receptor binding domain (RBD) of the spike protein, and ACE2, providing important information on the development of vaccines and small molecule or peptide inhibitors. In outpatients with COVID-19, neutralizing anti-SARS-CoV-2 monoclonal antibodies have been shown to reduce the incidence of disease progression and hospitalization when given within 5–7 days of the onset of the illness (Gupta A. et al., 2021; Dougan et al., 2021; Weinreich et al., 2021). One serious problem with using neutralizing antibodies to treat COVID-19 has been the rapid emergence and spread of mutations of the spike protein, which are not recognized by the available antibodies (Callaway, 2022). The NIH Guidelines (https://www.covid19treatmentguidelines.nih.gov/) indicate that almost all available antibodies have reduced activities against the B.1.1.529 (Omicron) variant of concern (VOC). Moreover, the neutralizing antibody Sotrovimab is no longer recommended as a treatment option for patients with COVID-19, since it has substantially reduced *in vitro* activity against the Omicron BA.2 subvariant, which has recently become one of the dominant subvariants in the United States and in Europe. Nonetheless, the FDA very recently has updated the Emergency Use Authorization (EUA) for Bebtelovimab, highlighting that this neutralizing monoclonal antibody retains activity against Omicron subvariants BA.2.12.1 and BA.4/BA.5.

An alternative approach to blocking the interaction between the SARS-CoV-2 and the ACE2 receptor is to use picomolar miniprotein inhibitors that have a high affinity for binding to the SARS-CoV-2 spike protein and compete with ACE2 binding. Two of these recently designed inhibitors were found to prevent infection in cultured cells more efficiently than the most potent monoclonal antibodies described to date (Cao et al., 2020).

The administration of a large amount of soluble ACE2 may neutralize the virus and slow viral entry into cells. Interestingly, a recombinant human soluble ACE2 (rhsACE2), corresponding to the extracellular domain of ACE2, was developed several years ago and found to be safe in healthy volunteers and in a small cohort of patients with acute respiratory distress syndrome (ARDS) in completed Phase I and Phase II clinical trials (*ClinicalTrials.gov identifier: NCT00886353*) (Haschke et al., 2013; Khan et al., 2017). This rhsACE2 was found to inhibit SARS-CoV-2 infection in cultured cells and in human blood vessel and kidney organoids (Monteil et al., 2020) and a clinical trial has been launched to use rhsACE2 to treat patients with COVID-19 (*ClinicalTrials.gov identifier: NCT04335136*). RhsACE2 has been successfully applied to COVID-19 patients (Zoufaly et al., 2020) and a number of variations of this approach have been described. For example, engineering of ACE2 N-glycosylation through site-directed mutagenesis or glycosidase treatment resulted in enhanced binding affinities and improved virus neutralization (Capraz et al., 2021). Treatment with recombinant human ACE2-Fc fusion protein (hACE2-Fc) effectively protected mice against SARS-CoV-2 infection: an advantage of this approach is that hACE2-Fc has a relative long half-life *in vivo* compared to soluble ACE2 (Zhang Z. et al., 2021).

A completely new approach to blocking virus entry into the cells is based on humoral innate immunity pattern recognition molecules. In particular, mannose-binding lectin (MBL), which binds the viral spike proteins, inhibited cell infection by SARS-CoV-2 variants of concern (VOCs), including Omicron (Stravalaci et al., 2022). A potential problem with the therapeutical application of MBL is the possibility that in advanced disease MBL may contribute to complement activation and result in uncontrolled inflammation.

TMPRSS2 protease inhibitors could be used to block a crucial step in the fusion of the virus with the cell membrane. The TMPRSS2 protease inhibitor, camostat mesylate, was reported to inhibit SARS-CoV-2 entry into lung cell lines (Hoffmann et al., 2020a). This drug has been approved in Japan and Korea to treat chronic pancreatitis and was repurposed to treat COVID-19 in a clinical trial (*ClinicalTrials.gov number NCT04353284*). Nafamostat mesylate, another drug that has been used for many years in Japan to treat acute pancreatitis and disseminated intravascular coagulation (DIC), was recently reported to inhibit SARS-CoV-2 infection in Calu3 human lung cells in the nanomolar range, with 10–15 fold higher efficiency than camostat mesylate (Hoffmann et al., 2020c; Yamamoto et al., 2020; Ko et al., 2021). The efficacy of nafamostat in COVID-19 patients is currently being evaluated in clinical trials (*ClinicalTrials.gov identifier: NCT04352400; Japan Registry of Clinical Trials: jRCTs031200026; Korea CRIS: KCT0005003*). It has been suggested that another TMPRSS2 inhibitor, bromhexine, presently used as a mucolytic cough suppressant, could be used to treat COVID-19 (Habtemariam et al., 2020; Maggio and Corsini, 2020). Novel TMPRSS2 inhibitors have recently been identified using high-throughput screening (Mantzourani et al., 2022), and a highly potent inhibitor has recently been well characterized (Shapira et al., 2022). This compound, N-0385, is a small peptidomimetic that is active at low doses (nanomolar) and inhibits SARS-CoV-2 infection in human lung cells and in donor-derived colon organoids. N-0385 blocks SARS-CoV-2 in different variants of concern (alpha, beta, gamma and delta) and is protective against infection and mortality in mice that express the human ACE2 receptor, when delivered as a nasal spray 12 h after infection.

Finally, since TMPRSS2 expression is controlled by androgens, which could explain the greater frequency of severe COVID-19 in males, it is possible that androgen receptor antagonists might reduce susceptibility to developing a serious COVID-19 infection (Stopsack et al., 2020). This possibility is supported by epidemiological studies that show that prostate cancer patients treated with anti-androgens are much less frequently affected by COVID-19 compared to patients who did not receive this treatment (Montopoli et al., 2020). This study is supported by new results from different laboratories and the effect of testosterone suppression in COVID-19 patients is being investigated in clinical trials, including a trial using Degarelix, an FDA-approved drug used to treat prostate cancer (*ClinicalTrials.gov identifier: NCT04397718*) (Wadman, 2020). However, a recent randomized phase 2 clinical trial did not find evidence that anti-androgen therapy had a therapeutic effect on COVID-19 patients (Welén et al., 2022).

## 3 Drugs that inhibit SARS-CoV-2 replication

Before the emergence of SARS-CoV-2 there was no specific antiviral treatment for coronavirus infections. Over the past 2 years considerable efforts have been directed towards identifying antivirals that are effective against SARS-CoV-2 and, in particular, toward repurposing of FDA-approved drugs that could become available much more quickly. So far, of the large number of antiviral drugs investigated in preclinical and clinical studies, three have been approved or have received emergency use authorization (EUA) from health authorities in different countries [https://www.bio.org/policy/human-health/vaccines-biodefense/coronavirus/pipeline-tracker]. As other antivirals used to treat RNA virus infections, these drugs target two key components of the virus replication machinery, the viral polymerase (remdesivir and molnupiravir) and the main viral protease (paxlovid), both of which are essential for viral replication.

### 3.1 Remdesivir

Remdesivir, an adenosine analog prodrug originally developed to treat the Ebola virus (EBOV), was found to inhibit the replication of human and animal coronaviruses *in vitro* and in preclinical studies (Eastman et al., 2020). Upon diffusion into the cell, remdesivir is metabolized into the nucleoside monophosphate form and ultimately into the active nucleoside triphosphate derivative, which is integrated into viral RNA by the viral RNA-dependent RNA polymerase (RdRp), resulting in chain termination (Eastman et al., 2020). Remdesivir was identified early as a promising candidate for treating COVID-19 because of its ability to inhibit SARS-CoV-2 *in vitro*, as well as in animal studies when treatment was initiated early during the course of infection (reviewed in Eastman et al.) (Eastman et al., 2020). These findings, along with the safety profile of remdesivir, as established in the clinical trial regarding Ebola virus (Eastman et al., 2020), supported the decision to evaluate remdesivir as a potential therapeutic drug to be repurposed for treating SARS-CoV-2 infections.

Initial observations of the clinical improvement in a limited number of patients who were seriously ill with COVID-19 and treated with remdesivir under compassionate use, were sufficiently encouraging to prompt the initiation of a large number of studies to investigate the effectiveness of remdesivir, alone or in combination with other drugs, against COVID-19 (see *ClinicalTrials.gov*). However, many of these studies were small and have produced conflicting results [reviewed in Vegivinti et al. (2022)].

No significant benefit was found in a randomized placebo-controlled trial of intravenous remdesivir conducted in China starting with 236 patients with COVID-19 (Wang Y. et al., 2020). On the other hand, on April 2020 the United States National Institute of Allergy and Infectious Diseases (NIAID) announced preliminary results from the Adaptive COVID-19 Treatment Trial (ACTT-1, NCT04280705), a double-blind, randomized, placebo-controlled phase 3 trial to evaluate the safety and efficacy of remdesivir in 1,062 adults hospitalized with COVID-19. The final report of the study concluded that remdesivir was superior to placebo in shortening patients’ time to recovery: a median of 10 days in hospital as compared to 15 days for those assigned to the placebo group (Beigel et al., 2020).

Based on these findings, on 1st May 2020 the FDA made remdesivir (VEKLURY^®^) available in the United States under an EUA to treat adults and children with severe COVID-19 disease (https://www.fda.gov/news-events/press-announcements/coronavirus-covid-19-update-fda-issues-emergency-use-authorization-potential-covid-19-treatment). The drug was then also authorized in the EU ( *https://www.ema.europa.eu/en/medicines/human/EPAR/veklury#authorisation-details-section* ). On 22nd October 2020, the FDA approved VEKLURY for use in adults and pediatric patients (12-years old and above) who required hospitalization (https://www.fda.gov/news-events/press-announcements/fda-approves-first-treatment-covid-19).

However, the mortality rate recorded in the ACTT-1 study though lower in the patients treated with remdesivir, remained high: 11.4% as compared to 15.2% in the placebo group on day 29 after enrollment (Beigel et al., 2020). Encouraging results regarding remdesivir-treated patients’ survival, including a comparative analysis of the Phase 3 SIMPLE-Severe trial and a real-world retrospective cohort of patients with severe COVID-19 (NCT04292899 and EUPAS34303) were reported in 2021 (Olender et al., 2021). In this analysis, by day 14 remdesivir treatment was associated with both an improvement in clinical recovery, and a 62% reduction in the risk of mortality compared with standard-of-care treatment.

On the other hand, in an open-label multinational study (NCT04292730) (Spinner et al., 2020) remdesivir was reported to be less effective in hospitalized patients with moderate COVID-19 pneumonia. In addition, the interim results of the WHO SOLIDARITY trial, a global, open-label, multicentric randomized four-arm trial comparing remdesivir, lopinavir/ritonavir, lopinavir/ritonavir with interferon beta-1a, and chloroquine or hydroxychloroquine (ISRCTN83971151/NCT04315948), concluded that not one of the four drugs produced any measurable benefits in terms of mortality or disease course (WHO Solidarity Trial Consortium et al., 2021). In the case of remdesivir, the study concluded that intravenous remdesivir had little or no effect on duration of hospital stay (the proportion still hospitalized on day 7, remdesivir vs*.* control was: 69% vs. 59%), or on mortality (301/2743 remdesivir vs*.* 303/2708 control).

In March 2022, however, the Canadian Treatments for COVID-19 (CATCO) trial (NCT04330690), a substudy of the global WHO Solidarity trial, concluded that with regard to the 1,282 patients admitted due to COVID-19, in-hospital mortality for patients treated with remdesivir was lower than for control patients: the 60-days mortality was 24.8% in the remdesivir arm, compared with 28.2% in the standard-of-care arm (RR 0.88, 95% CI 0.72–1.07) (Ali et al., 2022). In this study remdesivir treatment turned out to be especially beneficial in terms of preventing the need for mechanical ventilation [8.0% remdesivir vs*.* 15.0% standard of care (RR 0.53, 95% CI 0.38–0.75)], again suggesting a better outcome for patients with less severe disease (Ali et al., 2022).

The differing results from the clinical trials that used remdesivir on hospitalized patients, described above, likely contributed to growing uncertainty in the medical community regarding the effectiveness of the drug. However, the fact that most of the studies indicated that treatment with remdesivir is most successful when started in the early stages of infection, has created new perspectives. It should be noted that because remdesivir is administered as an infusion, treatment was until recently reserved only for hospitalized COVID-19 patients.

A recent placebo-controlled, randomized, double-blind trial that involved non-hospitalized COVID-19 patients who experienced symptom onset within the previous 7 days and presented at least one risk factor for disease progression, including age ≥60 years and obesity (PINETREE, NCT04501952) showed that a 3-days course of remdesivir had an acceptable safety profile and resulted in an 87% lower risk of hospitalization or death than placebo (Gottlieb et al., 2022). The results of this study were published during a surge in COVID-19 cases and the reduced susceptibility to several anti-SARS-CoV-2 monoclonal antibodies due to the Omicron variant. Thus, on 21st January 2022 the FDA authorized remdesivir for outpatient treatment for people at high risk of COVID-19 disease progression, and expanded the pediatric EUA to include treatment for non-hospitalized pediatric patients who are at high risk (https://www.fda.gov/news-events/press-announcements/fda-takes-actions-expand-use-treatment-outpatients-mild-moderate-covid-19).

In the United States, remdesivir is actually indicated for treating COVID-19 in adults and pediatric patients (12 years of age and older and weighing at least 40 kg) who are either hospitalized or not hospitalized and are at high risk of progression to severe COVID-19. Remdesivir is also authorized for these uses in pediatric patients below the age of 12, provided they weigh at least 3.5 kg. Detailed information can be found in the NIH COVID-19 Treatment Guidelines (https://www.covid19treatmentguidelines.nih.gov/antiviral-therapy/).

It should also be mentioned that remdesivir in combination with the JAK inhibitor baricitinib was found to be superior to remdesivir alone in reducing recovery time and accelerating improvement in clinical status among COVID-19 patients (NIAID ACTT-2 trial NCT04401579) (Kalil et al., 2021a). In a different study (ACTT-3 trial NCT04492475), on the other hand, remdesivir plus interferon beta-1a was, instead, not found to be superior to remdesivir alone in hospitalized patients with COVID-19 pneumonia; moreover, patients who required high-flow oxygen at baseline had worse outcomes after treatment with interferon beta-1a compared with those given placebo (Kalil et al., 2021b).

### 3.2 Molnupiravir

As discussed above, most studies indicate that antivirals such as remdesivir work best when given early in the course of infection, before severe disease occurs; since one major drawback of remdesivir is that the drug is administered as an infusion, the focus began to shift to oral drugs that could be used outside of hospital settings to treat mild illness, in order to prevent progression to severe disease. In late 2021, a different polymerase inhibitor, molnupiravir (Lagevrio), jointly developed by Merck and Ridgeback Biotherapeutics, became available as a pill.

Molnupiravir (MK-4482/EIDD-2801), β-D-N4-hydroxycytidine-5′-isopropyl ester, is a bioactive prodrug of β-D-N4-hydroxycytidine (NHC, EIDD-1931), an orally bioavailable ribonucleoside analogue originally described in 2003 and characterized by a broad-spectrum activity against RNA viruses, including influenza, the Ebola virus and several zoonotic coronaviruses (reviewed in Tian et al.) (Tian et al., 2022). In the case of SARS-CoV-2, molnupiravir inhibits virus replication in human lung tissue (Wahl et al., 2021), and blocks SARS-CoV-2 transmission in ferrets (Cox et al., 2021).

Molnupiravir, like remdesivir, is a nucleoside analogue, but the two drugs work in entirely different ways. Whereas remdesivir interferes with RNA chain elongation acting as a “chain terminator” (Eastman et al., 2020), molnupiravir acts as a mutagenizing agent that causes an “error catastrophe” during viral replication, thus hindering the formation of infectious viral particles (Kabinger et al., 2021).

A series of preclinical and clinical studies have indicated that molnupiravir is effective in the treatment of SARS-CoV-2 infection (Tian et al., 2022). After oral administration, molnupiravir is rapidly transformed into the active NHC metabolite in plasma, distributed to various organs, and converted into the NHC 5′-triphosphate by host kinases (Tian et al., 2022). Molnupiravir has been tested in several clinical trials, some of which are completed (https://clinicaltrials.gov/ct2/results?cond=COVID-19&term=molnupiravir).

The most informative evidence of the efficacy of molnupiravir in COVID-19 patients comes from the MOVe-OUT trial (NCT04575597), an international phase 2/3, double-blind, randomized, placebo-controlled trial, involving 1,433 patients with mild or moderate COVID-19, which started in October 2020. The trial evaluated the efficacy and safety of treatment with molnupiravir (800 mg twice daily for 5 days) started within 5 days of the onset of symptoms in non-hospitalized, unvaccinated adults with mild-to-moderate, laboratory-confirmed COVID-19 and at least one risk factor for severe COVID-19 illness. The interim results of the trial, announced by Merck in October 2021, found that the number of patients in the molnupiravir arm who died or needed to be hospitalized was approximately half the number of patients with such outcomes in the placebo arm. However, the recently published final results of the study, including all 1,433 participants, showed that hospitalization and deaths were approximately 30% lower in the molnupiravir group: for participants who received the drug the risk of death or hospitalization through day 29 was 6.8% (48 of 709 participants), as compared to 9.7% (68 of 699 participants) in the placebo arm (difference, −3.0 percentage points; 95% CI, −5.9 to −0.1) (Bernal et al., 2022). The proportion of patients who experienced adverse events was similar in the two groups.

The final results of the study, which were suggested to be linked to the emergence of the SARS-CoV-2 Delta variant—which had not yet become globally dominant during the first half of the trial—lowered expectations and limited the initial enthusiasm for the drug (Extance, 2022). Furthermore, even before the final trial results were released, concerns about molnupiravir’s mutagenic potential had been raised. Although animal tests indicated that the risk is low, laboratory tests suggested that there might be a risk of molnupiravir generating mutations in human DNA, especially in quickly reproducing cells such as blood cells or spermatozoa (Zhou et al., 2021; Extance, 2022).

In November 2021, the MHRA (Medicines and Healthcare products Regulatory Agency) in the United Kingdom approved the use of molnupiravir for at-risk patients with mild to moderate COVID-19, as the world’s first approved oral medication for SARS-CoV-2 (https://www.gov.uk/government/news/first-oral-antiviral-for-covid-19-lagevrio-molnupiravir-approved-by-mhra). On 23 December 2021 in the United States the FDA also granted molnupiravir an EUA for the treatment of mild to moderate COVID-19 in at-risk adults for whom alternative COVID-19 treatment options are not accessible or clinically appropriate (https://www.fda.gov/news-events/press-announcements/coronavirus-covid-19-update-fda-authorizes-additional-oral-antiviral-treatment-covid-19-certain). Following the FDA’s decision, the use of molnupiravir was also authorized in other countries; however, at the time of writing, the European Medicines Agency (EMA) has yet to grant conditional marketing authorization, while the Indian Council of Medical Research excluded molnupiravir from its COVID-19 treatment guidelines over toxicity concerns (Extance, 2022) on 13^th^ January 2022. Finally, in March 2022 WHO recommended the use of molnupiravir for non-severe COVID-19 patients with the highest risk of hospitalization only, including older and unvaccinated people, and patients with immunodeficiencies or chronic disease. Notably, WHO recommends that children and pregnant or breastfeeding patients should not be given molnupiravir, and that those who take it should have a contraceptive plan (https://www.who.int/news/item/03-03-2022-molnupiravir).

Several trials have recently been launched to establish the efficacy and safety of molnupiravir, including the PANORAMIC study in the United Kingdom (https://www.panoramictrial.org/), which is currently recruiting a large number of patients. Also, in March 2022 a pharmacovigilance program was launched by WHO in low- and middle-income countries to provide further evidence of molnupiravir’s safety in the general population (World Health Organization, 2022).

A day after molnupiravir was approved in the United Kingdom, Pfizer announced that its antiviral drug Paxlovid cut hospitalizations by 89%.

### 3.3 Paxlovid

Distinct from remdesivir and molnupiravir, which target the viral polymerase, paxlovid targets the highly conserved SARS-CoV-2 main protease (Mpro, also called 3CLpro), a three-domain chymotrypsin–like cysteine protease (Owen et al., 2021). Paxlovid is a co-packaged combination of nirmatrelvir (PF-07321332) and ritonavir tablets, developed for COVID-19 treatment and post-exposure prophylaxis. Nirmatrelvir is a peptidomimetic irreversible inhibitor of the SARS-CoV-2 Mpro, while ritonavir is a HIV-1 protease inhibitor and CYP3A inhibitor. As Nirmatrelvir is metabolized mainly by CYP3A4, coadministration of nirmatrelvir with a low dose (100 mg) of ritonavir, enhances nirmatrelvir pharmacokinetics which increases therapeutic benefit (Wen et al., 2022).

The SARS-CoV-2 genome encodes two polyproteins, pp1a and pp1ab, and four structural proteins (Owen et al., 2021). The polyproteins are cleaved by Mpro at multiple sites to generate a set of shorter, nonstructural proteins that are critical for viral RNA transcription and replication, including the RdRp complex (Wen et al., 2022). In addition to the key role that Mpro plays in viral replication, the lack of closely related homologs in humans, identifies Mpro as an attractive antiviral drug target (Wen et al., 2022). Based on early studies on the small molecule protease inhibitor PF-00835231, which was investigated to ascertain whether it could be used intravenously to treat SARS-CoV-1 (Boras et al., 2021), nirmatrelvir/PF-07321332 was recently discovered and characterized as an orally bioavailable SARS-CoV-2 Mpro inhibitor with *in vitro* pan-human coronavirus antiviral activity (Owen et al., 2021). Nirmatrelvir was also found to have good selectivity and safety profiles, as well as oral activity in a mouse-adapted SARS-CoV-2 model (Owen et al., 2021).

On 14th December 2021, Pfizer announced that paxlovid significantly reduced hospitalization and death, based on an interim analysis of the Phase 2/3 EPIC-HR trial (Evaluation of Protease Inhibition for COVID-19 in High-Risk Patients, NCT04960202), a randomized, double-blind study of non-hospitalized adult patients with COVID-19, who are at high risk of progressing to severe illness (https://www.pfizer.com/news/press-release/press-release-detail/pfizer-announces-additional-phase-23-study-results).

**FIGURE 2:**
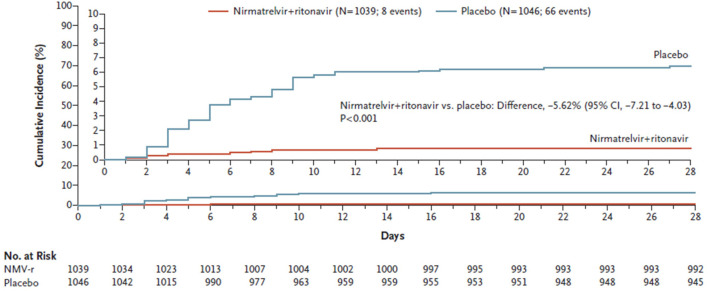
Cumulative percentage of patients with COVID-19-related hospitalization or death from any cause through day 28 among those treated within 5 days after symptom onset with nirmatrelvir plus ritonavir (Paxlovid) or placebo. (from Hammond et al., 2022).

The interim analysis showed an 89% reduction in the risk of COVID-19-related hospitalization or death from any cause compared to placebo in patients treated within 3 days of symptom onset. The final results of the study were published on 14th April 2022, and confirmed the interim results (Hammond et al., 2022) (Figure 2). A total of 2246 patients were assigned in a 1:1 ratio to receive either 300 mg of nirmatrelvir plus 100 mg of ritonavir or placebo twice daily for 5 days. Efficacy was maintained in the final analysis with a difference of −5.81 percentage points (95% CI, −7.78 to −3.84; *p* < 0.001; relative risk reduction, 88.9%). The viral load was lower with nirmatrelvir plus ritonavir than with placebo on day 5 of treatment, and all deaths reported occurred in the placebo group (Hammond et al., 2022). The incidence of adverse events that emerged during the treatment period was similar in the two groups; however, dysgeusia (5.6 vs 0.3%) and diarrhea (3.1 vs 1.6%) occurred more frequently with nirmatrelvir plus ritonavir than with placebo (Hammond et al., 2022).

One important consideration on the use of paxlovid is that the concomitant use of nirmatrelvir plus ritonavir and certain other drugs may result in potentially serious drug interactions; therefore paxlovid is contraindicated for patients already receiving certain drugs because of the risk of serious adverse events (Lamb, 2022) (Table 1). It should also be noted that the EPIC-HR trial was restricted to unvaccinated persons; a separate, ongoing phase 2/3 trial of nirmatrelvir plus ritonavir, EPIC-SR (EPIC-Standard Risk, NCT05011513) includes vaccinated persons. A third phase 2/3 EPIC-PEP trial (NCT05047601), to evaluate the efficacy and safety of paxlovid in preventing symptomatic SARS-CoV-2 infection in the adult household contacts of individuals with SARS-CoV-2 infection, is also currently ongoing.

**TABLE 1:** Main drug-drug interaction of nirmatrelvir/ritonavir (Paxlovid).

Paxlovid co-administration contraindicated/not recommended		
Amiodarone	Ergot derivatives	Pimozide
Apalutamide	Flecainamide	Primidone
Bosentan	Glecaprevir/pibrentasvir	Propafenone
Carbamazepine	Ivabradine	Quinidine
		Rifampin
Clopidrogrel	Lumacaftor/ivacaftor	Rifapentine
Clozapine	Lumateperone	Sildenafil for pulm hypertension
Disopyramide	Lurasidone	St John’s wort
Dofetilide	Meperidine	Tadalafil for pulm hypertension
Dronedarone	Midazolam (oral)	Tolvaptan
Enzalutamide	Phenobarbital	Vardenafil for pulm hypertension
Eplerenone	Phenytoin	Voclosporin
Paxlovid administration needs temporary withhold of concomitant drugs (if clinically appropriate)		
Alfuzosin	Estazolam	Rosuvastatin
Aliskiren	Everolimus	Salmeterol
Atorvastatin	Finerenone	Silodosin
Avanafil	Fibanserin	Simvastatin
Chemotherapy	Flurazepam	Sirolimus
Clonazepam	Lomitapide	Suvorexant
Clorazepate	Lovastatin	Tacrolimus
Colchicine	Naloxegol	Ticagrelor
Diazepam	Ranolazine	Triazolam
Eletriptan	Rimegepant	Ubrogepant
Erythromycin	Rivaroxaban	Vorapaxar
Paxlovid administration needs adjustment of concomitant medication dose and monitoring for adverse effect		
Alprazolam	Darifenacin	Pimavanserin
Amlodipine	Digoxin	Quetiapine
Apixaban	Elexacaftor	Rifabutin
Aripiprazole	Eluxadoline	Riociguat
Brexpiprazole	Fentanyl	Saxagliptin
Buspirone	Iloperidone	Sildenafil for erectile dysfunction
Cariprazine	Itraconazole	Ruxolitinib
Chlordiazepoxide	Ivacaftor	Tadalafil for erectile dysfunction
Cilostazol	Ketoconazole	Tamsulosin
Clarithromycin	Maraviroc	Tezacaftor
Clobazam	Mexiletine	Trazodone
Cyclosporine	Oxycodone	Vardenafil forerectile dysfunction

Modified from COVID-19 Treatment Guidelines Panel. Coronavirus Disease 2019 (COVID-19) Treatment Guidelines. National Institutes of Health. https://www.covid19treatmentguidelines.nih.gov/.

Paxlovid received its first EUA on the 22nd December 2021 in the United States for the treatment of mild-to-moderate COVID-19 in adults and pediatric patients (≥12 years of age and weighing ≥40 kg) who are at increased risk of progression to severe COVID-19. Paxlovid also received conditional authorization for the treatment of COVID-19 in the United Kingdom on the 31st December 2021, and more recently in the EU (January 2022) (Lamb, 2022).

In conclusion, in the last months, in addition to the first antiviral approved for COVID-19 treatment—remdesivir–two new antiviral drugs, molnupiravir and paxlovid, have received an EUA in different countries. Both drugs are available for oral use in non-hospitalized patients, but neither drug is a panacea: molnupiravir may cause mutations in human DNA, leading the health authorities in some countries to advise against its use during pregnancy, while other countries have chosen not to authorize it at all. And paxlovid’s possible interaction with a wide range of commonly used drugs limits its use.

A large number of antivirals that target the SARS-CoV-2 polymerase or the main protease are currently being developed. It is also expected that, as in the case of other viral diseases, such as AIDS and Hepatitis C, combinations of antivirals that target different viral or host proteins will be able to boost their effectiveness and reduce the risk of developing drug resistance (Schultz et al., 2022).

## 4 Non-steroidal anti-inflammatory drugs and COVID-19

As the incidence of COVID-19 began to rise in Europe, the French Health Minister, Olivier Véran, claimed that non-steroidal anti-inflammatory drugs (NSAIDs), like ibuprofen could aggravate the infection (Willsher, 2020), which led to a warning being published on the WHO and EMA websites. However, evidence has not emerged to substantiate this claim and the advisories have been taken down.

NSAIDs work by suppressing prostaglandin synthases 1 and 2, colloquially known as cyclooxygenase (COX)-1 and COX-2. These enzymes produce prostaglandins (PGs), lipids that can trigger pain and fever. COX-2 produces most of the PGs relevant to pain and inflammation. NSAIDs selective for inhibiting COX-2 include celecoxib, etoricoxib and diclofenac; ibuprofen is an NSAID that blocks both COXs.

The French Health Minister advised people to take paracetamol (acetaminophen) for fever instead of NSAIDs (Willsher, 2020). However, acetaminophen is an NSAID (Smyth et al., 2018). The most common oral daily dose - 1000 mg - inhibits PG formation by both COX-1 and COX-2 enzymes by about 50% (Catella-Lawson et al., 2001). Common daily doses of drugs like ibuprofen hit ∼100% at the time of peak action (Catella-Lawson et al., 2001).

Acetaminophen and other NSAIDs reduce body temperature the same way - by inhibiting the central PGE_2_ dependent activation of EPr3 (Ushikubi et al., 1998). They are also analgesic through the same mechanism, by reducing PGE_2_-dependent central and peripheral activation of EPrs (Grosser et al., 2017). One must move up the dose response curve with NSAIDs to achieve maximal PG inhibition (as is achieved with common daily doses of other NSAIDs that inhibit both COXs like ibuprofen) to gain anti-inflammatory efficacy. Thus, at acetaminophen 3–4000 mg/day, there is a similar GI (García Rodríguez and Hernández-Díaz, 2001) and hypertensive (Sudano et al., 2010) adverse effect profile as with other NSAIDs.

However, acetaminophen has a particular risk of hepatotoxicity at higher doses, which are avoided for that reason. The makers of acetaminophen made a virtue of necessity and marketed acetaminophen as an anti-pyretic, analgesic. They claimed that it was not an NSAID because it did not cause GI toxicity. At the time (before the discovery of COX-2), all NSAIDs competed in direct-to-consumer advertising in the United States by claiming a safer GI profile, so, the myth that acetaminophen was not an NSAID was marketed and widely believed. Like other NSAIDs, acetaminophen has PG independent effects of unestablished relevance to their clinical profile. Most commonly, we also use aspirin, another NSAID, at doses that are not anti-inflammatory and take advantage of its particular action on the platelets at low doses, thereby minimizing its GI toxicity.

Given the thrombotic complications of COVID-19, it was suggested that aspirin might be beneficial in treating COVID-19. Thrombotic events appear to be no more common in patients with Acute Respiratory Distress Syndrome caused by COVID-19 than by other viral or bacterial causes. The RECOVERY Collaborative Group has shown that in patients hospitalized with COVID-19, aspirin was not associated with reductions in 28-days mortality or in the risk of progressing to invasive mechanical ventilation or death but was associated with a small increase in the rate of being discharged alive within 28 days (RECOVERY Collaborative Group, 2022). Ongoing trials are assessing the potential utility of low-dose aspirin in delaying or postponing hospitalization in patients with milder disease.

Membrane sphingolipids (van der Meer-Janssen et al., 2010) and membrane cholesterol (Hu et al., 2019) modulate viral entry into cells. Furthermore, the activation of phospholipases by viral attachment to its cellular receptors releases many bioactive lipids, including PGs, such as PGE_2_, PGD_2_, and prostacyclin (PGI_2_), which can both promote and restrain inflammation (Theken et al., 2021).

For example, the infection of certain immune cells (microglia) with a related coronavirus (not the one that causes COVID-19) activates a proinflammatory response (the inflammasome) to combat the pathogen; however, PGD_2_ increases the expression of PYDC3, a putative inflammasome inhibitor, in certain immune cells in mice (Vijay et al., 2017). The SARS coronavirus responsible for the 2003 outbreak directly binds to the COX-2 promotor and increases its expression (Yan et al., 2006), boosting PG production capacity. There is also evidence that PGE_2_ inhibits SARS coronavirus replication (Sander et al., 2017). Indomethacin, an NSAID, blocks coronavirus RNA synthesis, but independently of COX inhibition (Amici et al., 2006). In contrast, COX-2–dependent PGE_2_ attenuates the chronic antiviral lymphocyte response of unresolved viral infection (Schaeuble et al., 2019). Based on these findings, multiple contrasting possibilities are plausible, but evidence has yet to emerge of the relevance of these observations to the course or treatment of COVID-19.

Perhaps the most provocative finding relates to PGD_2_, the predominant COX-2 product of mast cells. It acts through its two receptors, DPr1 and DPr2. DPr1 signaling delays the migration of dendritic cells (DCs) to the lung and lymph nodes by down-regulating the expression of C-C chemokine receptor type 7 (CCR7) on respiratory DCs in response to infection. DPr1 inhibition enhances DC migration and, in turn, T cell proliferation, which increased survival in older, but not younger mice after SARS-CoV infection (Theken and FitzGerald, 2021). More recently, DPr1 deletion or blockade with an antagonist, asapiprant, or the deletion of an upstream biosynthetic enzyme–the phospholipase PLA2G2D–protected middle-aged mice against lethal infection with SARS-CoV-2 (Zhao et al., 2011). While asapiprant is being investigated in ongoing clinical trials, other studies are exploring the spectrum of the lipidomic response to infection in search of predictive signatures and therapeutic opportunity (Roy Wong et al., 2022).

Patterns of individual PG formation may turn out to reflect the intensity of the disease and forecast its course but also to signal the opportunity to intervene with potentially preventative therapies before patients progress to severe disease. For example, microangiopathy and hemostatic activation are features of severe COVID-19 and roughly 30% of patients have elevated d-dimers at hospitalization. To give just one example, thromboxane (Tx) biosynthesis is markedly elevated in patients with acute respiratory distress syndrome (ARDS) and preclinical studies have shown that Tx receptor (TPr) antagonism prevents the evolution of a lipopolysaccharide (LPS)-induced syndrome of ARDS in sheep (Kühl et al., 1988). Unlike NSAIDs, which suppress the vasodilator PGs that maintain renal blood flow (RBF) in syndromes such as ARDS, TPr antagonism would be expected to sustain RBF even in renoprival syndromes, such as ARDS, where NSAIDs cannot be used (Qi et al., 2002). Thus, serial analysis of PGs in patients with COVID-19 may suggest that the modulation of individual PGs be considered for therapeutic intervention or as biomarkers that are predictive of disease progression. Interestingly, *ex vivo* studies of monocyte-derived macrophages, which are themselves strongly implicated in ARDS pathogenesis, suggest that even mild infections with COVID-19 inflict a lasting proinflammatory eicosanoid signature, which remains evident 1 month after infection (Bohnacker et al., 2022).

### 4.1 Summary

If there is no clear evidence of risk regarding NSAIDs, should patients with clinically complicated SARS-CoV-2 infections receive them? No. There is no evidence of benefit, either. If such a patient also had poor kidney function, maintenance of renal blood flow would become critically dependent on vasodilator PGs, such as PGE_2_ and PGI_2_. This situation might also predispose the patient to the gastrointestinal and cardiovascular complications of NSAIDs. However, until we have robust evidence, patients who are in chronic pain should continue to take their NSAIDs, rather than turning to opiates. Given that the elderly are an at-risk group for severe COVID-19, an association between NSAIDs and the disease may merely reflect reverse causality. Low-dose aspirin appears to be minimally effective in patients hospitalized with COVID-19. Its utility in patients with milder disease remains to be seen.

## 5 Corticosteroids

The place of corticosteroids in the treatment of COVID-19 has been first established by the outcome of the RECOVERY trial (RECOVERY Collaborative Group Horby et al., 2021). This was a randomized, controlled, open-label, adaptive, platform trial that compared a range of possible treatments with usual care in patients hospitalized with COVID-19. Around 10% of all hospitalized patients with COVID-19 in the United Kingdom were enrolled in the trial, and the control arm fatality rate was consistent with the overall case fatality rate for hospitalized patients with COVID-19 in the United Kingdom (RECOVERY Collaborative Group Horby et al., 2021).

Prior to this trial there was considerable confusion about the place of steroids in the treatment for severe viral infections. On the one hand, slower clearance of viral RNA has been observed in patients with SARS, MERS and influenza who were treated with systemic corticosteroids. On the other, steroids offered a theoretical benefit after the viral replication phase, when immunopathology is dominant. However, prior to the RECOVERY trial, no clinical trials of sufficient size and rigor had been performed in such settings.

In this trial, 6 mg of dexamethasone, given once daily for up to 10 days, was compared to usual care alone (RECOVERY Collaborative Group Horby et al., 2021). The primary outcome was 28-days mortality. In contrast to SARS and MERS, the viral replication phase in COVID-19 is early after infection, and then declines.

**FIGURE 3:**
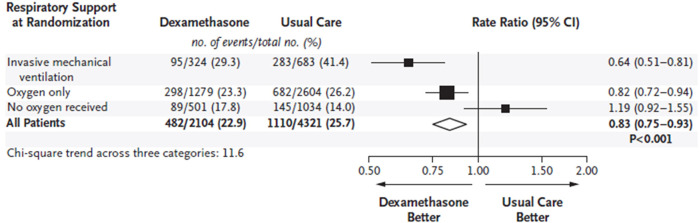
Effect of dexamethasone on 28-days mortality according to respiratory support at the time of randomization in hospitalized patients with COVID-19. Rate ratios are plotted as squares, with the size of each square proportional to the amount of statistical information that was available. Horizontal lines represent 95% confidence intervals. (from RECOVERY Collaborative Group Horby et al., 2021).

As reported (RECOVERY Collaborative Group Horby et al., 2021), 2104 patients randomly allocated to receive dexamethasone were compared with 4,321 patients who were concurrently allocated to usual care. Overall, 482 (22.9%) patients allocated to the dexamethasone group, and 1,110 (25.7%) patients allocated to usual care died within 28 days (age adjusted rate ratio [RR], 0.83; 95% confidence interval [CI], 0.75 to 0.93; *p* < 0.001). Based on sub-group analysis, the proportional and absolute mortality rate reductions varied significantly depending on the level of respiratory support at randomization: dexamethasone reduced deaths by one-third in patients who received invasive mechanical ventilation (29.3 vs 41.4%; RR, 0.64; 95% CI, 0.51–0.81), by one-fifth in patients who received oxygen without invasive mechanical ventilation (23.3 vs 26.2%; RR, 0.82; 95% CI, 0.72–0.94), but had no significant protective effect on those who did not receive any respiratory support (RECOVERY Collaborative Group Horby et al., 2021) (Figure 3).

Most deaths were due to COVID-19, and these deaths were less frequent in the dexamethasone group than in the usual care group. The very small number of reported serious adverse reactions consisted of the recognized adverse effects of glucocorticoids (RECOVERY Collaborative Group Horby et al., 2021).

Despite some methodologic caveats about the platform design of RECOVERY (Normand, 2021), this trial provides clear evidence that treatment with 6 mg of dexamethasone, once daily for up to 10 days, reduces 28-days mortality in patients with COVID-19 who are receiving respiratory support. Based on these results, one death would be prevented by treatment of around eight patients who require invasive mechanical ventilation, or around 34 patients who require oxygen without invasive mechanical ventilation.

A cautionary note must be sounded regarding the possibility of harm to those patients who did not require respiratory support at the time of randomization. For these patients, mortality was higher for those who received dexamethasone (17.8 vs. 14.0%) although the difference did not attain statistical significance (RR, 1.19; 95% CI, 0.92–1.55).

The usefulness of dexamethasone for patients with severe pulmonary complications following COVID-19 infection has been supported by further investigations (Tomazini et al., 2020; WHO REACT Working Group Sterne et al., 2020). In particular, a subsequent meta-analysis of seven trials involving glucocorticoids (dexamethasone, hydrocortisone, or methylprednisolone) for critically ill patients with COVID-19, including RECOVERY, has confirmed the findings of this trial (WHO REACT Working Group Sterne et al., 2020).

Based on this evidence, the use of dexamethasone on hospitalized patients requiring respiratory support is widely recommended. Details on specific recommendations for or against corticosteroid therapy are provided by several treatment guidelines (COVID-19 Treatment Guidelines Panel, 2022; Bhimraj et al., 2022).

## 6 Immunomodulatory drugs

Severe COVID-19 is characterized by interstitial pneumonia/acute respiratory distress syndrome and systemic inflammation, with elevated levels of proinflammatory cytokines, such as interleukin-1 (IL-1), IL-6 and tumor necrosis factor alpha 1. Increased serum levels of IL-6 were found to predict adverse outcomes, especially the need for mechanical ventilation, and mortality (Del Valle et al., 2020; Herold et al., 2020). Several observational studies and randomized controlled trials (RCTs) have therefore targeted IL-6 and its downstream signaling, such as the JAK and signal transducer and activator of transcription (STAT) pathway.

Most therapeutic strategies have to date focused on the inhibition of the IL-6 receptor using the monoclonal antibody tocilizumab, which had already been approved for treating rheumatologic diseases and cytokine release syndrome induced by chimeric antigen receptor therapy. In observational studies performed in the United States and globally, tocilizumab appeared to improve clinical outcomes for hospitalized patients with COVID-19 pneumonia (Morrison et al., 2020; Xu et al., 2020; Gupta S. et al., 2021). However, the initial RCTs that examined this monoclonal antibody led to conflicting results (Stone et al., 2020; Hermine et al., 2021; Rosas et al., 2021; Salvarani et al., 2021). Many of these clinical trials appear, though, to be constrained by their small size, heterogeneous patient populations, and/or low frequency of concomitant administration of corticosteroids, which are now used as the standard of care for patients with severe COVID-19. The two largest RCTs to evaluate tocilizumab - REMAP-CAP and RECOVERY - both reported a survival benefit for tocilizumab in certain COVID-19 patients when used on background corticosteroid therapy. In particular, the multiplatform, adaptive REMAP-CAP trial showed that in critically ill COVID-19 patients who received organ support in the intensive care unit (ICU), treatment with tocilizumab markedly improved outcomes, in terms of the number of days free from organ support and in-hospital mortality, compared to standard care, which included glucocorticoids in the majority of patients (>80%) (REMAP-CAP Investigators et al., 2021). The open-label, platform RECOVERY trial found that among 4,116 hospitalized COVID-19 patients with hypoxia and systemic inflammation, adding tocilizumab to standard care significantly reduced the primary outcome of 28-days mortality, compared to standard care alone. The finding that consistent results were obtained in patients who received systemic glucocorticoid treatment at randomization (82%), suggests that the benefits of tocilizumab were additional to those of glucocorticoids (RECOVERY Collaborative Group, 2021a).

Based on available evidence from these RCTs, on 24th June 2021, the FDA issued an EUA for the use of tocilizumab in combination with corticosteroids on hospitalized adult and pediatric patients (2 years of age or older) with COVID-19 who require supplemental oxygen, non-invasive or invasive mechanical ventilation, or extracorporeal membrane oxygenation.

Sarilumab is the second most commonly studied monoclonal antibody blocking IL-6 receptor in COVID-19 patients. Parallel with encouraging data on tocilizumab, early uncontrolled studies that tested sarilumab also produced promising results (Benucci et al., 2020; Gremese et al., 2020), leading to the initiation of several RCTs. An adaptive design trial involving 420 patients with severe or critical COVID-19 found that sarilumab did not meet its primary endpoint of time to improvement by two or more points on an ordinal seven-point clinical status scale compared to placebo (Lescure et al., 2021). Likewise, there was no significant difference between groups regarding the key secondary endpoint, the proportion of patients alive on day 29 (Lescure et al., 2021). A similar adaptive trial showed that in 298 critically ill COVID-19 patients who required mechanical ventilation, sarilumab failed to exhibit any benefits over placebo with regards to the primary endpoint - the proportion of patients with a ≥1 point improvement in clinical status on day 22 (Sivapalasingam et al., 2022). Among critical patients who received mechanical ventilation and corticosteroids at baseline, there was a numerical but not significant reduction in the risk of mortality with sarilumab compared to placebo (Sivapalasingam et al., 2022). In the REMAP-CAP trial, the efficacy results for sarilumab were similar to those for tocilizumab. Compared to patients randomized to standard of care, those allocated to sarilumab had more organ support-free days and a greater likelihood of survival during hospitalization (REMAP-CAP Investigators et al., 2021). In summary, sarilumab had a favorable effect on survival in patients with severe COVID-19 pneumonia in one RCT, while it was neutral in two other trials.

Siltuximab is a monoclonal antibody that prevents IL-6 from binding to its receptors. A non-peer- reviewed study found that intravenous administration of siltuximab for 30 COVID-19 patients who required ventilator support was associated with a significantly lower mortality rate compared to that reported for control patients who received standard care alone (Gritti et al., 2021).

JAK inhibitors exert immunomodulatory effects by inhibiting the STAT-mediated signaling pathways of several cytokines. Baricitinib, specifically, is an orally administered inhibitor of JAK1 and JAK2 that blocks the intracellular signaling pathways of cytokines, which are known to be elevated in severe COVID-19, including IL-2, IL-6, interferon-γ and granulocyte-macrophage colony-stimulating factor. Moreover, unlike other JAK inhibitors, baricitinib was also thought to inhibit AP2-associated protein kinase 1, a pivotal regulator of clathrin-dependent endocytosis, thereby preventing viral entry into target cells (Stebbing et al., 2020). This evidence, along with promising results from early observational studies that tested baricitinib treatment for hospitalized patients with COVID-19 (Cantini et al., 2020; Titanji et al., 2021), prompted the initiation of RCTs. The ACTT-2 trial found that for 1,033 patients with COVID-19 pneumonia who received supplemental oxygen, high-flow oxygen or noninvasive ventilation, the combination of baricitinib and remdesivir was superior to remdesivir alone in terms of the primary outcome of time to recovery, as measured on an 8-category ordinary scale (Kalil et al., 2021a). The combination treatment group also had 30% higher odds of improvement in terms of clinical status on day 15 than the control group (Kalil et al., 2021a). The COV-BARRIER trial showed that for 1525 COVID-19 patients who did not require mechanical ventilation, and who had at least one elevated inflammatory marker, treatment with baricitinib in addition to standard of care (which predominantly included corticosteroids) did not reduce the incidence of primary composite endpoint of progression to high-flow oxygen, noninvasive ventilation, invasive mechanical ventilation or mortality compared to standard of care alone (Marconi et al., 2021). However, treatment with baricitinib reduced the key secondary outcome of mortality by 38.2% within 28 days (Marconi et al., 2021). Taken together, the results of these trials suggest that baricitinib may have additive or even synergistic effects with standards of care, including remdesivir and corticosteroids. Based on evidence from RCTs, baricitinib received EUA from the FDA for the treatment of severe COVID-19, in combination with remdesivir, in November 2020, and then as monotherapy in July 2021. In May 2022 the FDA finally approved baricitinib for the treatment of adult patients hospitalized with COVID-19 who required supplemental oxygen, non-invasive or invasive mechanical ventilation, or extracorporeal membrane oxygenation (Rubin, 2022).

Results from a RCT that examined tofacitinib, a selective inhibitor of JAK1 and JAK3, in the setting of COVID-19, have also been reported. The STOP-COVID trial found that for 289 patients hospitalized with COVID-19 pneumonia, tofacitinib was superior to placebo in reducing the incidence of the primary endpoint of mortality or respiratory failure within 28 days (Guimaraes et al., 2021). These effects were consistent regardless of the duration of symptoms and use of glucocorticoids at baseline (Guimaraes et al., 2021).

Clinical trials involving several JAK inhibitors for the treatment of COVID-19 are ongoing, and their results will hopefully provide valuable information on the usefulness of these agents.

## 7 Complement inhibitors

The complement system is one of the host immune system’s first lines of defense against invading pathogens (Noris and Remuzzi, 2013). However, its potentially beneficial role in providing immunity to SARS-CoV-2 has been called into question by multiple lines of evidence that implicate uncontrolled complement activation in the pathogenesis of severe COVID-19 (Noris and Remuzzi, 2013; Fodil and Annane, 2021; Noris, 2021; Afzali et al., 2022). Mechanistically, SARS-CoV-2 can directly or indirectly activate all the three complement pathways (i.e., the classical, alternative and lectin pathways) (Holter et al., 2020; Yu et al., 2020; Ali et al., 2021). This process leads to the formation of several effectors, including the terminal products C5a and C5b-9, which contribute to lung inflammation and injury, endothelial damage and dysfunction, with the inflammatory response eventually spreading to the circulation and other organs (Noris, 2021). Indeed, in patients with COVID-19, plasma levels of C5a and soluble C5b-9 increase in proportion to disease severity (Carvelli et al., 2020; Holter et al., 2020). Furthermore, extensive complement deposition has been reported in the lung and kidney tissues of patients who died of COVID-19 (Magro et al., 2020; Diao et al., 2021).

Based on this background, several uncontrolled studies and some controlled clinical trials using different complement inhibitors have already been performed, involving patients with severe COVID-19, while others are underway. To date, most of the strategies used to target complement activation in COVID-19 have focused on C5 inhibition, in particular using the monoclonal antibody eculizumab, which has already been approved for the treatment of paroxysmal nocturnal hemoglobinuria, atypical hemolytic uremic syndrome, myasthenia gravis and neuromyelitis optica spectrum disorder. Initial case series and explorative studies found that patients with severe COVID-19 who were treated with eculizumab, including in combination with the JAK1/2 inhibitor ruxolitinib (Diurno et al., 2020; Giudice et al., 2020; Laurence et al., 2020; Mastellos et al., 2020), experienced more positive clinical outcomes. A pilot study involving five critically ill COVID-19 patients who were on mechanical ventilation and received eculizumab documented a mortality rate of 50%, compared to estimates as high as 97% for similar patients given standard care alone during the same period (Pitts, 2021). In a non-randomized controlled study involving 80 patients with severe COVID-19 who were admitted to an ICU, treatment with eculizumab with more frequent and higher dosing compared to what is indicated for atypical hemolytic uremic syndrome appeared to improve 15-days survival compared to standard therapy alone. However, serious infectious complications and ventilator-associated pneumonia were two-fold more common in eculizumab-treated patients than in controls (Annane et al., 2020). More recently, another non-randomized controlled study showed that adding two 900 mg doses of eculizumab to standard therapy for ten patients with severe COVID-19, who were receiving continuous positive airway pressure support for ≤ 24 h, safely improved respiratory dysfunction and reduced the risk of reaching the combined endpoint of mortality, or discharge with chronic complications, compared to 65 contemporary similar controls who were given standard therapy alone (Ruggenenti et al., 2021).

On the other hand, a phase III randomized controlled trial (RCT) with ravulizumab, another anti-C5 monoclonal antibody with a prolonged half-life compared to eculizumab, (NCT04369469), that involved patients with severe COVID-19 who required mechanical ventilation has been stopped after an interim analysis revealed a lack of efficacy (Covid-19 roundup, 2021).

Ongoing RCTs that target C5 in severe COVID-19 are evaluating the monoclonal antibodies eculizumab (NCT04346797) and ravulizumab (NCT04570397 and NCT04390464), and peptide inhibitors of C5, such as zilucoplan (NCT04382755).

The impact of blocking the C5a-C5aR1 axis is also under clinical investigation. An initial phase II open-label RCT tested the blockade of C5a with the monoclonal antibody vilobelimab in 30 patients with severe COVID-19. Although there was no significant difference between patients randomized to vilobelimab and those allocated to standard of care alone in terms of the primary endpoint of change in the ratio of arterial oxygen tension over fraction of inspired oxygen (PaO2/FiO2) on day five, there was a trend toward improved survival in the anti-C5a treatment group (Vlaar et al., 2020). This adaptive design trial has now progressed to a phase III placebo-controlled stage (NCT04333420) involving 399 patients who require mechanical ventilation, with 28-days mortality as the primary endpoint. Similarly, a large phase II/III RCT (NCT04449588) to test the anti-C5a antibody BDB-001 on patients with severe COVID-19 is ongoing. Despite the promising results from C5aR1 blockade with the monoclonal antibody avdoralimab in a mouse model of acute lung injury (Casadevall and Pirofski, 2020), where it decreased pulmonary neutrophil and macrophage infiltration, treatment with avdoralimab in a double-blind RCT in patients with severe COVID-19 pneumonia, did not meet its primary endpoint of improving clinical status over placebo on days 14 or 28 (Carvelli et al., 2022).

The inhibition of C3, which is upstream of C5 in the complement cascade, does not appear to be a safe approach for patients with COVID-19, since it could reduce the antiviral response and prevent immunity to other infectious diseases. Nonetheless, the C3 inhibitor AMY-101 was initially used in two case series to treat four COVID-19 patients with acute respiratory distress syndrome, all of whom eventually recovered (Mastaglio et al., 2020; Mastellos et al., 2020). A larger phase II RCT involving 144 patients with acute respiratory distress syndrome due to COVID-19 has been planned (NCT04395456) but is not yet recruiting.

Other strategies that aim to target the early stages of complement activation involve the inhibition of C1 esterase or the mannose-binding lectin-associated serine proteases (MASPs) of the lectin pathway. In a preliminary case series involving five patients with severe COVID-19 pneumonia, the administration of the human recombinant C1 esterase inhibitor conestat alpha was found to be safe and associated with clinical improvements, with all patients being discharged from hospital within 3 weeks (Urwyler et al., 2020). These encouraging results promoted the initiation of two similar phase II RCTs that aimed to evaluate whether adding conestat alpha to standard therapy for hospitalized COVID-19 patients could reduce the risk of progression to severe disease compared to standard therapy alone (NCT04530136 and NCT04414631). The use of the anti-MASP2 antibody narsoplimab was initially reported in six patients with severe COVID-19 and acute respiratory distress syndrome, all of whom subsequently recovered and were discharged from hospital (Rambaldi et al., 2020). A phase II adaptive platform trial designed to evaluate rapidly promising investigational agents, including narsoplimab, to reduce the time to recovery or mortality risk in critically ill COVID-19 patients, is currently underway (NCT04488081).

Collectively, early clinical findings and emerging clinical trial evidence suggest that some complement inhibitors may have therapeutic benefits in severe COVID-19. The results of ongoing phase II/III clinical trials will hopefully elucidate the benefit-to-risk profile of complement inhibitors and clarify the optimal target(s) in the complement cascade.

## 8 Anticoagulants and other antithrombotic agents

### 8.1 Pathophysiologic and pharmacologic rationale

Microvascular and macrovascular thrombotic complications, including arterial but especially venous thromboembolism (VTE), appear to be common clinical features of COVID-19, particularly in hospitalized and critically ill patients (Nadkarni et al., 2020; Piazza et al., 2020). These thrombotic/thromboembolic events are promoted by the inflammatory process underlying viral infections such as SARS-CoV-2. In particular, inflammation induces the excessive production of thrombin and a reduction in fibrinolysis caused by endothelial dysfunction due to the ongoing viral infection (Subramaniam and Scharrer, 2018). Moreover, the hypoxia that characterizes SARS-CoV-2 infection also contributes to thrombosis by enhancing blood viscosity (Subramaniam and Scharrer, 2018).

Heparin is a glycosaminoglycan with anticoagulant activity produced by basophils and mast cells in all mammals. It activates antithrombin III which, in turn, inhibits thrombin (Factor II), Factor X and other proteases involved in the blood coagulation cascade (Gray et al., 2012). Heparin and low-molecular-weight heparins (derived from unfractionated heparin by depolymerization) are commonly used prophylactically to prevent post-surgical venous thromboembolism, as well as in non-surgical patients with heart failure or acute respiratory failure, conditions characterized by reduced mobility. They are also used in the pharmacological treatment of deep vein thrombosis, pulmonary embolism and acute coronary syndromes.

### 8.2 Preclinical evidence of efficacy

Heparin also exhibits anti-inflammatory properties that could be relevant in the context of COVID-19. According to the immune-thrombosis model, the formation of thrombi inside blood vessels, in particular in microvessels, induces an innate immune response (Engelmann and Massberg, 2013). Thus, blocking thrombin with heparin may dampen the inflammatory response. Heparin also elicits anti-inflammatory functions through mechanisms that are independent of its anticoagulant activity, which include binding to inflammatory cytokines, inhibiting neutrophil chemotaxis and leukocyte migration, neutralizing the positively charged peptide complement factor C5a, and sequestering acute phase proteins (Poterucha et al., 2017). In an animal model of acute lung injury, treatment with nebulized heparin reduced injury-mediated coagulation factors and inflammation in the alveolar space, without affecting systemic coagulation (Camprubí-Rimblas et al., 2020). Heparin also appears to protect the vascular endothelium. Apart from pathogens, histones released from damaged cells can also cause endothelial injury (Xu et al., 2009). Heparin can antagonize histones, thereby protecting endothelial cells (Zhu et al., 2019). This protective function seems to extend to the endothelial tight junctions as demonstrated in a sepsis animal model, where unfractionated heparin reduced lung edema and vascular leakage (Liu et al., 2019). Finally, experimental evidence suggests that heparin could have antiviral potential. Indeed, the structure of heparin highly resembles heparan sulfate, a linear polyanionic polysaccharide used by a large number of human viruses, including coronaviruses, to attach to target cells (Milewska et al., 2014). A recent study has used spectroscopic techniques along with molecular modeling to show that the SARS-CoV-2 Spike S1 protein receptor binding domain interacts with heparin (Mycroft-West et al., 2020). This observation raises the intriguing possibility that heparin could compete with heparan sulfate to bind to SARS-CoV-2, thereby preventing virus entry into cells. However, this hypothesis remains to be addressed. The antiplatelet agent dipyridamole was found to suppress SARS-CoV-2 replication *in vitro* at concentrations comparable to those reported in the blood of patients treated with this medication after ischemic stroke (Liu X. et al., 2020).

### 8.3 Clinical evidence of efficacy in patients with COVID-19

A systematic review estimated the VTE event rate to be about 17% among COVID-19 inpatients, increasing to around 28% for those admitted to the ICUs (Jiménez et al., 2021). Several organizations have released guidelines regarding the prevention and management of VTE in patients with COVID-19. All agree that hospitalized, non-pregnant patients with COVID-19 should receive, at a minimum, a prophylactic dose of anticoagulants to prevent VTE (Barnes et al., 2020; Marietta et al., 2020; Thachil et al., 2020; American Society of Hematology, 2021; National Institute for Health Care Excellence, 2021). Nevertheless, the optimal antithrombotic strategy across the spectrum of COVID-19 severity remains ill defined. Many RCTs have been performed and others are ongoing to evaluate the efficacy and safety of a variety of antithrombotic regimens in COVID-19 patients during all phases of the illness, from the community to hospital admission, when critically ill, and after hospital discharge.

The ACTIV-4B RCT showed that in 657 outpatients with COVID-19, treatment with aspirin, prophylactic or therapeutic doses of apixaban (a selective inhibitor of factor Xa) compared to placebo did not reduce the rate of the primary composite endpoint of mortality, symptomatic venous or arterial thromboembolism, myocardial infarction, stroke, or hospitalization for cardiovascular or pulmonary causes (Connors et al., 2021). However, the study was terminated when only 9% of the planned total number of participants could be enrolled due to the lower than anticipated primary event rates. At present, routine administration of thromboprophylaxis is not recommended for ambulatory COVID-19 patients.

Several RCTs have been conducted to evaluate the role of therapeutic doses of heparin in reducing VTE events, disease progression or mortality in hospitalized patients with COVID-19 who do not require ICU-level care. An international, multiplatform RCT that combined data from the ATTACC, REMAP-CAP and ACTIV-4A studies showed that among 2219 moderately ill COVID-19 patients, a therapeutic dose of anticoagulation with unfractionated or low-molecular-weight heparin was more effective than usual care thromboprophylaxis with regards to the primary outcome of survival free from organ support (ATTACC, ACTIV-4a and REMAP-CAP Investigators et al., 2021). Major bleeding occurred in 1.9% of patients who received therapeutic dose anticoagulation and in 0.9% of those given thromboprophylaxis (ATTACC, ACTIV-4a and REMAP-CAP Investigators et al., 2021). The RAPID trial found that among 465 moderately ill COVID-19 patients with increased D-dimer levels, therapeutic compared to prophylactic anticoagulation treatment with unfractionated or low-molecular-weight heparin did not significantly reduce the primary composite outcome of non-invasive or invasive mechanical ventilation, ICU admission, or mortality up to 28 days (Sholzberg et al., 2021). However, therapeutic dose anticoagulation was associated with a decrease in the secondary outcome of all-cause mortality, and there was no increase in major bleeding compared to prophylactic anticoagulation (Spyropoulos et al., 2021). The HEP-COVID trial showed that among 253 hospitalized COVID-19 patients with elevated D-dimer levels or a high sepsis-induced coagulopathy score, most of whom (67%) did not require an ICU-level of care, therapeutic dose low-molecular-weight heparin significantly reduced the primary composite outcome of thromboembolism or death compared to standard prophylactic or intermediate dose heparins (Spyropoulos et al., 2021). There was no meaningful difference in terms of major bleeding between groups, even though confidence intervals were wide (Spyropoulos et al., 2021).

Together, the available evidence from RCTs supports the hypothesis that therapeutic anticoagulation with low-molecular-weight heparin or unfractionated heparin is associated with improved outcomes for hospitalized patients with COVID-19 who are not critically ill or in the ICU setting, particularly for those with elevated D-dimer levels. The beneficial effect of therapeutic anticoagulation in moderately ill COVID-19 patients using heparin does not seem to extend to other classes of anticoagulant agents. The ACTION trial showed that in 615 hospitalized patients with COVID-19 and elevated D-dimer levels, therapeutic anticoagulation with the factor Xa inhibitor rivaroxiban (and with enoxaparin in the small number of clinically unstable patients) did not reduce the primary composite endpoint of death, duration of hospitalization, or duration of oxygen use compared to prophylactic anticoagulation with heparin but did increase the risk of bleeding (Lopes et al., 2021a). Therefore, the use of therapeutic doses of rivaroxiban or other direct oral anticoagulants is not recommended for hospitalized COVID-19 patients.

The role of therapeutic doses of heparin in reducing VTE events, disease progression and mortality has also been investigated in hospitalized patients who require ICU-level care. The ATTACC, REMAP-CAP and ACTIV-4A multiplatform trial showed that in critically ill patients, therapeutic dose anticoagulation with unfractionated or low-molecular-weight heparin did not improve the primary outcome of survival free from organ support compared to usual care thromboprophylaxis, and was associated with more major bleeding events (REMAP-CAP, ACTIV-4a, ATTACC Investigators et al., 2021). The INSPIRATION trial found that in 562 COVID-19 patients admitted to the ICU, intermediate dose (1 mg/kg enoxaparin daily) compared to standard dose (40 mg enoxaparin daily) thromboprophylaxis did not reduce the primary composite outcome of venous or arterial thrombosis, treatment with extracorporeal membrane oxygenation, or mortality within 30 days (INSPIRATION Investigators et al., 2021). Major bleeding occurred in 2.5% of patients in the intermediate dose group and in 1.4% of those in the standard dose group (INSPIRATION Investigators et al., 2021).

Thus, current evidence from RCTs supports the use of standard dose thromboprophylaxis in critically ill patients with COVID-19. In this patient population, therapeutic dose heparin is only indicated for documented thromboembolic complications (Flaczyk et al., 2020; Cuker et al., 2021). It is not known why therapeutic doses of heparin appear to improve clinical outcomes in moderately ill, but not in critically ill, COVID-19 patients. It is conceivable that the disease may be too advanced in patients requiring ICU-level care or organ support for them to benefit from therapeutic heparin, with organized thrombi that are quite resistant to the action of antithrombin III, the endogenous anticoagulant potentiated by heparin (Ten Cate, 2021).

There is no consensus yet on the role that extended thromboprophylaxis plays beyond a patient’s hospital stay. Recently, the MICHELLE trial has showed that for 320 patients at high risk who were discharged after COVID-19 hospitalization, thromboprophylaxis with rivaroxiban for 35 days–compared to no extended anticoagulation–significantly reduced the primary composite outcome of venous thromboembolic events (these included events that were either symptomatic or detected using routine imaging tests, and arterial thrombotic events and cardiovascular death) without increasing the risk of major bleeding (Ramacciotti et al., 2022).

Since platelets play a central role in the pathogenesis of COVID-19, the use of antiplatelet agents is also under clinical investigation. The platelet aggregation inhibitor dipyridamole in particular may have additional antiviral properties (Aliter and Al-Horani, 2021), and three small RCTs are evaluating its effects on hospitalized patients with COVID-19 (NCT04391179, NCT04424901 and NCT04410328).

The results of ongoing clinical trials will hopefully clarify the role of pre-hospital and post-discharge antithrombotic prophylaxis for COVID-19 patients, and the potential benefits of other prophylactic and therapeutic agents, such as antiplatelet drugs.

## 9 Drugs that are not recommended because of proven lack of efficacy

### 9.1 Hydroxychloroquine/chloroquine

Despite *in vitro* evidence (Liu J. et al., 2020; Fantini et al., 2020) and anecdotal reports, there is no evidence of efficacy for the use of chloroquine or hydroxychloroquine, either alone or with azithromycin, for the treatment of COVID-19 patients according to the NIH COVID-19 Treatment Guidelines (https://www.covid19treatmentguidelines.nih.gov/) (COVID-19 Treatment Guidelines Panel, 2022) (Cavalcanti et al., 2020; Geleris et al., 2020; RECOVERY Collaborative Group Horby et al., 2020; Self et al., 2020; WHO Solidarity Trial Consortium et al., 2021). Similarly, current trials on hydroxychloroquine/chloroquine for the prophylaxis of SARS-CoV-2 infection (https://clinicaltrials.gov/ct2/results?cond=COVID19&term=preexposure+prophylaxis&cntry=&state=&city=&dist=) are not supportive, and therefore the NIH Panel recommends against the use of hydroxychloroquine/chloroquine prophylaxis for COVID-19.

### 9.2 Ivermectin

This FDA-approved antiparasitic drug has demonstrated a degree of efficacy *in vitro* against SARS-CoV-2 infection (Caly et al., 2020; Yang et al., 2020), and uncontrolled, interventional studies have reported a degree of efficacy or no benefits (Amhed et al., 2020; Chachar et al., 2020; Khan et al., 2020). However, the NIH Panel indicates that there is insufficient evidence for COVID-19 treatment and recommended against its use (COVID-19 Treatment Guidelines Panel, 2022).

### 9.3 Fluvoxamine

Fluvoxamine is a selective serotonin reuptake inhibitor, cytochrome P450 inhibitor and regulator of autophagy, which is approved by the FDA for the treatment of obsessive-compulsive disorder as well as depression. Based on the anti-inflammatory effects of fluvoxamine that have been documented in preclinical studies (Rafiee et al., 2016; Rosen et al., 2019), trials involving humans are exploring the possibility of using this drug to treat COVID-19. Several clinical studies (NCT04668950; NCT04718480; NCT04342663; NCT04510194) are still in progress, while the one that has been completed failed to show any significant benefits. The TOGETHER trial, an adaptive platform, supported a double-blind randomized placebo-controlled trial involving non-hospitalized adults with COVID-19 with a known risk factor for severe illness, which showed there was a lower risk of the primary composite outcome of retention in the emergency department for >6 h or admission to a tertiary hospital for fluvoxamine-treated patients than in the placebo group (Reis et al., 2022). However, there was no significant difference between mortality rates for the two study groups. This finding is consistent with the recent results of a phase 3, double-blind, randomized, placebo-controlled trial that tested the efficacy of fluvoxamine (and also metformin and ivermectin) in preventing serious SARS-CoV-2 infection in 1,431 nonhospitalized adult patients, enrolled early after the onset of COVID-19 symptoms (Bramante et al., 2022). Fluvoxamine, as well as metformin and ivermectin failed to prevent the occurrence of hypoxemia, an emergency department visit, hospitalization, or death associated with COVID-19 (the primary composite endpoint).

### 9.4 Lopinavir/ritonavir

Like other HIV protease inhibitors (darunavir/cobicistat), lopinavir/ritonavir have been used in clinical trials (RECOVERY Collaborative Group, 2020; WHO Solidarity Trial Consortium et al., 2021), but the NIH Panel recommends against their use in its guidelines for the treatment of COVID-19 in hospitalized, as well as non-hospitalized, patients due to insufficient evidence of efficacy (COVID-19 Treatment Guidelines Panel, 2022).

### 9.5 Colchicine

Used as an anti-inflammatory drug for a variety of conditions (Van Echteld et al., 2014), the NIH Panel guidelines recommend against its use to treat hospitalized and non-hospitalized COVID-19 patients (COVID-19 Treatment Guidelines Panel, 2022). Indeed, in the large, randomized RECOVERY trial involving hospitalized COVID-19 patients, colchicine did not exhibit benefits in terms of 28-days mortality or other secondary outcomes (RECOVERY Collaborative Group, 2021b). Similarly, for non-hospitalized patients with COVID-19 in the large, randomized, placebo-controlled COLCORONA trial, colchicine failed to reach the primary endpoint of reducing hospitalizations and deaths (Tardif et al., 2021).

### 9.6 Interferons

Approved to treat hepatitis B and hepatitis C virus infections, interferons are currently being tested in clinical trials. However, the NIH Panel recommends against the use of systemic interferon beta for the treatment of hospitalized patients with COVID-19, against the use of interferon alfa or lambda for the treatment of hospitalized patients with COVID-19, except within a RCT, as well as against the use of interferons for the treatment of non-hospitalized patients with mild or moderate COVID-19, except within a RCT (COVID-19 Treatment Guidelines Panel, 2022). These recommendations are supported by recent trials showing that interferon beta-1a was not of clinical benefit in the treatment of hospitalized patients with COVID-19, either alone or in combination with remdesivir or corticosteroids (WHO Solidarity Trial Consortium et al., 2021).

### 9.7 Convalescent plasma

Plasma from patients who have recovered from COVID-19 is not recommended by the NIH Panel (COVID-19 Treatment Guidelines Panel, 2022). While this is due to insufficient evidence of its efficacy, the treatment has also been superseded by the use of monoclonal antibodies.

### 9.8 Lactoferrin

Lactoferrin, or lactotransferrin, is an iron-binding glycoprotein used in clinical trials (NCT04526821; NCT04412395; NCT04475120; NCT04847791) for its immune-regulatory effects. There is no evidence of its efficacy against COVID-19 (Algahtani et al., 2021).

## 10 Drugs with insufficient evidence to recommend for or against

Several drugs are still being tested in clinical trials (for the latest update see: www.ClinicalTrials.gov) and, considering the preliminary results there is insufficient evidence to recommend for or against them (90). Here are some examples.

### 10.1 Nitazoxanide

This FDA-approved, broad-spectrum thiazolide antiparasitic agent, and its metabolite, tizoxanide, exhibit *in vitro* activity against a number of viral infections, including MERS-CoV, SARS-CoV, SARS-CoV-2 (Rossignol, 2014; Rocco et al., 2021). On the basis of early clinical trials (Rocco et al., 2021), however, the NIH Panel did not find the evidence sufficient to recommend nitazoxanide for the treatment of COVID-19 (COVID-19 Treatment Guidelines Panel, 2022). On the other hand, a recent multicenter, randomized, double-blind, placebo controlled trial in 405 patients hospitalized with COVID-19 pneumonia found that nitazoxanide did not prevent ICU admission compared to placebo (Rocco et al., 2022). However, treatment with nitazoxanide may accelerate symptom resolution, shorten duration of oxygen therapy, and reduce levels of inflammatory mediators (Rocco et al., 2022).

### 10.2 Granulocyte-macrophage colony-stimulating factor (GM-CSF) inhibitors

Granulocyte-macrophage colony-stimulating factor (GM-CSF) is an FDA-approved myelopoietic growth factor and proinflammatory cytokine, FDA-approved, that plays a central role in a broad range of immune-mediated diseases (Hamilton, 2002). Anti-GM-CSF monoclonal antibodies may limit inflammation by minimizing the production of several proinflammatory mediators involved in COVID-19 (De Luca et al., 2020). Several clinical trials have been completed (NCT04411680; NCT04326920; NCT04707664) or are still open (NCT04569877; NCT04341116), with inconclusive results. Preliminary data published in the preprint format and a small, randomized trial with anti-GM-CSF monoclonal antibodies provided conflicting results (Patel et al., 2020; Cremer et al., 2021; Temesgen et al., 2022). Lenzilumab produced a significant improvement in the ventilator-free survival through day 28 for COVID-19 patients compared to patients treated with placebo (Temesgen et al., 2022). Other studies, however, did not report a survival benefit for otilimab (Patel et al., 2020) or mavrilimumab (Cremer et al., 2021) compared to placebo. Thus, the NIH COVID-19 Treatment Guidelines Panel states that there is insufficient evidence for the treatment of hospitalized COVID-19 patients (COVID-19 Treatment Guidelines Panel, 2022).

### 10.3 Anakinra

Anakinra, an interleukin-1 receptor antagonist, is used to treat Multisystem Inflammatory Syndrome in Children. The COVID-19 Treatment Guidelines Panel has found that there is insufficient evidence to recommend either for or against the use of anakinra for treating COVID-19, except in RCTs. Indeed, while the SAVE-MORE trial, involving hospitalized patients with moderate or severe COVID-19 pneumonia, reported a lower risk of clinical progression of the illness in patients given anakinra than in those who received placebo (Kyriazopoulou et al., 2021), the REMAP-CAP, an open-label, adaptive platform, randomized controlled trial, showed that anakinra did not have any efficacy in reducing the combined endpoint of in-hospital mortality and days on organ support (The REMAP-CAP Investigators, 2021).

### 10.4 Vitamin C and D

Vitamin C and D have been used as COVID-19 therapy and prophylaxis. The COVID-19 Treatment Guidelines Panel has concluded that there is insufficient evidence to recommend either for or against the use of vitamin C for the treatment of COVID-19 in non-critically and critically ill patients (COVID-19 Treatment Guidelines Panel, 2022). There are no controlled trials that have definitely demonstrated that there are any clinical benefits regarding the use of vitamin C in ambulatory or critically ill patients with COVID-19, and the available observational data are inconclusive (Zhang J. et al., 2021; Thomas et al., 2021). Similarly, the few randomized clinical trials of vitamin D in patients with moderate to severe COVID-19, preclude robust conclusions regarding the effectiveness of this treatment on major disease outcomes (Murai et al., 2021).

## 11 Cell-based therapy

### 11.1 Mesenchymal stromal cells and the rationale for their use in COVID-19

In recent years, stem cells have attracted much attention, and they are generally believed to have the potential to treat several diseases (Najar et al., 2022).

Compared to embryonic stem cells, Mesenchymal stromal cells (MSCs) are more easily available, more ethical to work with, and easy to freeze and thaw under standard conditions *in vitro*, making clinical application more convenient and safer (Shivakumar et al., 2016). MSCs have low immunogenicity and possess homing properties and have been shown to modulate overactive immune and hyperinflammatory processes, promote tissue repair, and secure antimicrobial molecules (Caplan and Correa, 2011; Perico et al., 2018; Thompson et al., 2020; Alagesan et al., 2022; Najar et al., 2022). MSCs have been reported to limit inflammation and fibrosis in the lungs (Harrell et al., 2019) and are being studied in acute respiratory distress syndrome (ARDS) of viral (Chen et al., 2020) and non-viral etiology (Matthay et al., 2019; Yip et al., 2020).

Together, these observations present the rationale for hypothesizing that MSCs could reduce the acute lung injury and inhibit the cell-mediated inflammatory response induced by SARS-CoV-2 (Sadeghi et al., 2020; Cai et al., 2021). Notably, because MSCs lack the ACE2 receptor for SARS-CoV-2 entry into host cells, they are themselves resistant to the infection (Lukomska et al., 2019).

### 11.2 Clinical evidence of efficacy in patients with COVID-19

Data supporting cell-based therapy with MSCs in COVID-19 patients are limited to small, open-label studies and a small number of randomized controlled trials.

Initial pilot studies on intravenous infusion of bone marrow-derived- or umbilical cord-derived MSCs (hUC-MSCs) for hospitalized COVID-19 patients with severe illness were performed in China. They consistently reported that MSC treatment was safe and accelerated pulmonary function recovery compared to standard of care therapies (Leng et al., 2020; Shu et al., 2020; Feng et al., 2021). Some confirmatory evidence has been obtained in small studies (Iglesias et al., 2021).

Notably, in an uncontrolled observational cohort of 210 severely/critically ill COVID-19 patients, significantly higher survival rates were reported in those who received hUC-MSC infusion before intubation (Ercelen et al., 2021).

There have been few RCTs. In a double-blind, phase 1/2a trial involving COVID-19 patients with acute respiratory distress syndrome, subjects were randomized to either hUC-MSCs treatment (*n* = 12, 2 cell infusions) or placebo (also two infusions of vehicle solution), both in addition to best standard of care treatment (Lanzoni et al., 2021). The levels of inflammatory cytokines decreased significantly in hUC-MSC- treated subjects by day 6. Moreover, cell treatment had significantly improved patients’ survival by day 31, compared to the placebo group. Despite the reported benefits of UC-MSC infusion in this study, the interpretation of the results is again limited by the small sample size and a change in the eligibility criterion–from enrolling only individuals who were on invasive mechanical ventilation to including those who were receiving high-flow oxygen or non-invasive ventilation. Another randomized controlled trial involving critically ill patients with COVID-19, albeit still with a small sample size (*n* = 40), showed that the survival rate for those given a single intravenous infusion of hUC-MSCs was 2.5 times higher than that in the control group. However, the length of stay in the intensive care unit (ICU) and ventilator usage were comparable for the two groups (Dilogo et al., 2021). Less encouraging are recent results from the STROMA-CoV-2 study, a multicenter, double-blind, randomized, placebo-controlled trial involving adult patients with SARS-CoV-2-induced early mild-to-severe ARDS (Monsel et al., 2022). Although the three hUC-MSC infusions were not associated with any serious adverse events during treatment or thereafter (until day 28), changes in the partial pressure of oxygen to fractional inspired oxygen (PaO2/FiO2)-ratio between baseline and day 7 post-infusion did not differ significantly in the hUC-MSC *versus* placebo group.

The above are all short-term studies. As part of a previous UC-MSC clinical trial, the long-term consequences of this cell treatment have recently been reported in a prospective, longitudinal, randomized, double-blind, placebo-controlled phase 2 trial, in which 100 COVID-19 patients were followed up at 3-months intervals for 1 year (Shi et al., 2022). Interestingly, 17.9% of patients in the UC-MSC group had normal lung CT images at month 12, but none in the placebo group, indicating that UC-MSC administration may confer long-term benefits in terms of recovery from lung lesions and symptoms for COVID-19 patients.

As of November 2021, according to Clinicaltrials.gov (https://www.clinicaltrials.gov), a total of 74 clinical trials were being assessing MSCs for COVID-19 treatment. Among these MSC clinical trials, 22 were using hUC-MSCs, 15 adipose tissue-derived MSCs (AD-MSCs) and 11 were using bone marrow-derived-MSCs (BM-MSCs).

In summary, while investigations into the clinical use of MSCs to treat COVID-19 remain in the preliminary stages, they could potentially be used in future treatments. Nonetheless, so far, no MSC products have been approved by the FDA for treating COVID-19. Therefore, the NIH COVID-19 Treatment Guidelines Panel recommends against the use of MSCs to treat COVID-19, except in an approved RCT (COVID-19 Treatment Guidelines Panel, 2022).

## 12 Concomitant medications for underlying conditions in COVID-19 patients

Individuals with underlying chronic conditions, such as cardiovascular disease (CVD), pulmonary disease, diabetes, and malignancies are at higher risk of severe illness with COVID-19. These patients are usually prescribed medications to treat these disorders. Early in the pandemic, some of these agents, such as angiotensin-converting enzyme inhibitors (ACEi), angiotensin receptor blockers (ARBs) (Bavishia et al., 2021), HMG-CoA reductase inhibitors (statins) (Lee K. C. H. et al., 2020; INSPIRATION-S Investigators, 2022), and H-2 receptor antagonists (Mather et al., 2020), were reported to be potential COVID-19 therapeutic agents or, in some cases, to present potential hazards.

A predictable consequence of the discovery that membrane-bound ACE2 is the functional receptor through which SARS-CoV-2 enters human cells was the concern that ACEi and ARBs, through the up-regulation of the expression of ACE2, may contribute to adverse outcomes related to COVID-19 (Lopes et al., 2021b). Currently, following on at least three randomized clinical trials, there is no evidence that discontinuing renin-angiotensin system inhibitors for underlying medical conditions offers a clinical benefit for patients with COVID-19 (Bauer et al., 2021; Lopes et al., 2021b; Cohen et al., 2021). The American Heart Association, the American College of Cardiology, and the Heart Failure Society of America issued a joint statement that renin-angiotensin-aldosterone system antagonists, such as ACEi and ARBs, should be continued as prescribed for patients with COVID-19 (Bozkurt et al., 2020).

Although simvastatin has been reported to downregulate the SARS-CoV-2-induced inflammatory response and to impair viral infection through the disruption of lipid rafts (Teixeira et al., 2022), a large retrospective cohort study conducted by the United States Veterans Health Administration found that statin use was associated with lower odds of 30-days mortality, both in subjects with and without a positive respiratory swab for SARS-CoV-2, indicating that statins may not have beneficial effects that are COVID-19-specific (Wander et al., 2022).

As discussed above, NSAIDs were postulated to have a negative impact without a clear mechanistic explanation (see Section 4 of this document) (Yousefifard et al., 2020). However, after reviewing the evidence, the FDA stated that there is no evidence linking the use of NSAIDs to worsening COVID-19 and advised patients to use them as directed (Food and Drug Administration, 2020).

According to the United States National Institutes of Health (NIH) COVID-19 Treatment Guidelines Panel, patients with COVID-19 who are treated with concomitant medications for an underlying medical condition should not discontinue these medications during the acute management of COVID-19, unless discontinuation is otherwise warranted by their clinical condition (COVID-19 Treatment Guidelines Panel, 2022).

The same Panel recommends against using medications off-label to treat COVID-19 if they have not been shown to be safe and effective for this indication in a RCT (COVID-19 Treatment Guidelines Panel, 2022).

Finally, when prescribing medications to treat COVID-19, clinicians should always assess the patient’s current medications for potential drug-drug interactions and/or additive adverse effects (COVID-19 Treatment Guidelines Panel, 2022).

## 13 Treatment management for children with multisystem inflammatory syndrome (MIS-C)

In April 2020, during the peak of the COVID-19 pandemic in Europe, multisystem inflammatory syndrome in children (MIS-C) was first described in reports of children presenting with a severe multisystem hyperinflammatory illness that was temporally associated with a preceding SARS-CoV-2 infection or exposure (Toubiana et al., 2020; Verdoni et al., 2020). Since then, cases have been reported worldwide and by the end of June 2021, the Centers for Disease Control and Prevention (CDC) has reported 4,196 confirmed MIS-C cases in United States, and 37 deaths (Nijman et al., 2020; Centers for Disease Control and Prevention, 2021a; Antúnez-Montes et al., 2021). Most MIS-C patients have serologic evidence of previous SARS-CoV-2 infection, but only a minority are RT-PCR positive for SARS-CoV-2 at presentation (Dufort et al., 2020; Feldstein et al., 2020). In published case series, many of the pediatric patients with this hyperinflammatory syndrome are described as having had a fever and mucocutaneous manifestations similar to those of Kawasaki’s disease, a rare form of vasculitis found in children that can cause coronary artery aneurysm (McCrindle et al., 2017; Riphagen et al., 2020; Toubiana et al., 2020; Verdoni et al., 2020). Some patients have presented with features of toxic shock syndrome, secondary hemophagocytic lymphohistiocystosis, or macrophage activation syndrome (Crayne and Cron, 2019). Although the cause of Kawasaki’s disease remains unknown, a preceding or active infection has been suspected (Benseler et al., 2005). Like Kawasaki disease, MIS-C is a syndrome with a range of clinical presentations and no pathognomonic findings or diagnostic tests. Unlike Kawasaki disease, however, MIS-C has been suggested in early reports to predominantly affect adolescents and children above the age of 5 years and to be associated with more frequent cardiovascular involvement (Belhadjer et al., 2020; Riphagen et al., 2020; Verdoni et al., 2020). The current CDC case definition for MIS-C includes, 1) an individual aged < 21 years presenting with fever (>38 °C), laboratory evidence of inflammation, and evidence of clinically severe illness that requires hospitalization with multisystem (>2) organ involvement; and 2) no alternative plausible diagnoses; and 3) positive for current or recent SARS-CoV-2 infection as ascertained with a RT-PCR, antigen test, or serology results; or COVID-19 exposure within the 4 weeks prior to the onset of symptoms (Centers for Disease Control and Prevention, 2021b).

Nonetheless, the pathogenesis of MIS-C is still being elucidated. Differences have been reported between MIS-C and typical Kawasaki disease in terms of cytokine expression and the elevation of inflammatory markers. Moreover, differences in cytokine expression (tumor necrosis factor alpha and interleukin-10) have been shown between MIS-C and acute COVID-19 in children (Diorio et al., 2020; Rowley et al., 2020). However, given the reported clinical similarity between MIS-C and Kawasaki disease, the approach to treating MIS-C has been similar to that for Kawasaki disease. Thus, in cohorts of children with MIS-C the most commonly used therapeutic approach is intravenous immunoglobulin (IVIG) and glucocorticoids (Belhadjer et al., 2020; Lee P. Y. et al., 2020; Dufort et al., 2020; Feldstein et al., 2020; Pouletty et al., 2020; Toubiana et al., 2020; Verdoni et al., 2020; Whittaker et al., 2020; Feldstein et al., 2021). The IVIG in combination with glucocorticoids is also the recommendation of the American College of Rheumatology for the first-level treatment for most hospitalized children with MIS-C (Henderson et al., 2022). Several non-randomized studies indicate that the front-line combination of IVIG/glucocorticoids results in less treatment failure, faster recovery of cardiac function, shorter ICU stays, and a decreased need for treatment escalation compared to IVIG monotherapy (Belhadjer et al., 2020; Jonat et al., 2021; McArdle et al., 2021; Ouldali et al., 2021; Son et al., 2021). On this basis, the recommendation is that IVIG be used in combination with low-to-moderate dose glucocorticoids for children hospitalized with MIS-C, but not the routine use of IVIG monotherapy, unless glucocorticoid therapy is contraindicated (COVID-19 Treatment Guidelines Panel, 2022). Indeed, there is uncertainty regarding the use of glucocorticoid monotherapy *versus* IVIG plus glucocorticoids as initial therapy for MIS-C because comparative studies evaluating these two treatment regimens have not been performed. On the other hand, there is insufficient evidence to recommend either for or against the use of glucocorticoid monotherapy for children with MIS-C (COVID-19 Treatment Guidelines Panel, 2022).

The combination of IVIG/glucocorticoid therapy usually results in a clinical improvement within the first 24 h, characterized by the resolution of fever, improvement in organ function, and reduced levels of inflammatory markers, particularly C-reactive protein. Should MIS-C be refractory to combined treatment (persistent fever, worsening organ dysfunction, and inflammatory marker increase), intensification therapy with higher-dose glucocorticoids (Chiotos et al., 2020; Son et al., 2021) or the IL-1 receptor antagonist anakinra (Lee P. Y. et al., 2020; Feldstein et al., 2020; Pouletty et al., 2020), or the monoclonal antibody anti-TNFα infliximab, have been proposed. However, no comparative studies evaluating intensification therapies for children with refractory MIS-C have been conducted yet, making it impossible to determine which of these agents is more effective in this setting. Infliximab plus IVIG has been tested in a single-center retrospective cohort study as initial immunomodulatory therapy for 72 children with MIS-C (Cole et al., 2021). The patients treated with infliximab plus IVIG were less likely to require additional therapy compared to those treated with IVIG alone, and had a shorter ICU stay, decreased left ventricular dysfunction, and more rapid decline in C-reactive protein levels. Notably, children with MIS-C who receive multiple immunomodulatory agents are at risk of infection and need to be monitored carefully.

Similarly to children with Kawasaki disease, it is expected that platelet activation and endothelial dysfunction may also occur in those with MIS-C (McCrindle et al., 2017). Thus, there is agreement that children who have MIS-C should also be given low-dose aspirin if they are not at risk of bleeding despite the absence of evidence from RCTs. However, again based on evidence from Kawasaki disease patients, children with MIS-C who have large coronary artery aneurysms or with moderate-to-severe left ventricular dysfunction (and are at risk of intracardiac thrombosis) should receive therapeutic anticoagulation, unless contraindicated due to bleeding risk factors (COVID-19 Treatment Guidelines Panel, 2022). Given the uncertainty of the benefit and the risk of major bleeding (Whitworth et al., 2021), prophylactic or therapeutic anticoagulation for children with MIS-C, but without large coronary artery aneurysms or left ventricular dysfunction, should be evaluated on a case-by-case basis, taking the risk factors for thrombosis into account.

## 14 Conclusion

**FIGURE 4:**
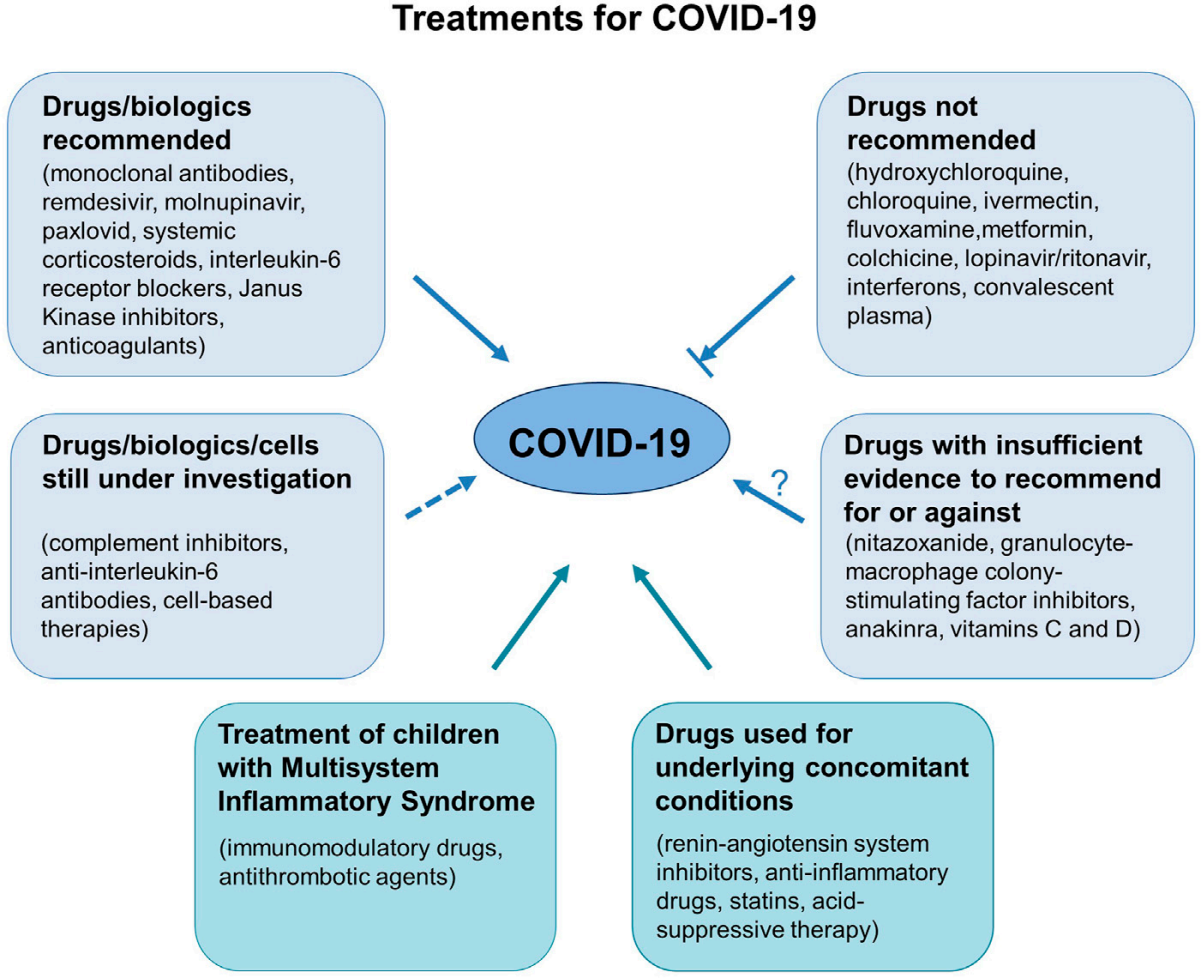
Summary of treatment spectrum for COVID-19. The panel shows drugs/biologics recommended or not recommended for treating patients with COVID-19. There are also medicines for which there is insufficient evidence to recommend for or against their use in these patients, as well as drugs/biologics and cell-based therapies that are under investigation. Concomitant medications used to treat underlying conditions in COVID-19 patients should also be taken into consideration for safety reasons. Managing treatment for children with Multisystem inflammatory syndrome, an illness that is temporally associated with preceding SARS-CoV-2 exposure is also mentioned.

Over the past 2 years the scientific community has made impressive efforts and advances, which are unparalleled in modern history, to understand the nature and pathophysiologic mechanisms underlying SARS-CoV-2 infection and the wide spectrum of the clinical manifestations of the associated disease, COVID-19. This facilitated and accelerated the race to develop new disease-specific drugs/biologics and to explore the possibility of repurposing medicines that are already available. The initial focus has been on severely ill, hospitalized patients. Now attention is turning to limiting or halting the progression of the illness at the onset of symptoms at home. Observational studies worldwide provided the rationale for conducting randomized clinical trials, and results from these formed the backbone for recommendations for or against particular treatments by the health authorities and panels of international experts. They also informed conclusions regarding whether given medicines have sufficient evidence for authorities to recommend for or against their use (Figure 4). However, much effort was wasted on small, under powered studies unable ever to provide a definitive answer. It has been estimated that this was true of more than 90% of the studies performed in the United States (Bugin and Woodcock, 2021). Several new agents and treatments are also still under investigation, so these recommendations will be periodically updated. Overall, a large spectrum of effective and safe new drugs/biologics are now available for treating COVID-19. Nonetheless, the access to the novel therapies (much like the access to vaccines) is limited to wealthy nations that can afford them, while they remain unavailable or scarce in low- and middle-income countries due to their high costs and restricted supply chains. The global nature of the COVID-19 pandemic demands that we overcome these inequalities, and rapidly find ways for global health organizations, pharmaceutical companies, and national/regional health authorities to cooperate. This is not just a moral imperative, but also essential to counteract the generation and circulation of SARS-CoV-2 variants, restricting the possibility of the virus making a sustained comeback in countries attempting to eradicate SARS-CoV-2 infection.
